# Untargeted Metabolomic Biomarker Discovery for the Detection of Ectopic Pregnancy

**DOI:** 10.3390/ijms251910333

**Published:** 2024-09-26

**Authors:** Onur Turkoglu, Ayse Citil, Ceren Katar, Ismail Mert, Robert A. Quinn, Ray O. Bahado-Singh, Stewart F. Graham

**Affiliations:** 1 Department of Obstetrics and Gynecology, Maternal Fetal Medicine Division, Baylor College of Medicine, Houston, TX 77030, USA; 2 Department of Obstetrics and Gynecology, Zekai Tahir Burak Women’s Health Education and Research Hospital, Ankara 06230, Turkey; 3 Department of Obstetrics and Gynecology, Division of Gynecological Oncology, Advocate Health, Chicago, IL 60642, USA; 4 Department of Biochemistry, Michigan State University, Lansing, MI 48824, USA; 5 Department of Obstetrics and Gynecology, Oakland University William Beaumont School of Medicine, Corewell Health, William Beaumont University Hospital, Royal Oak, MI 48073, USA; 6 Metabolomics Department, Corewell Health Research Institute, William Beaumont University Hospital, Royal Oak, MI 48073, USA

**Keywords:** ectopic pregnancy, gynecology, metabolomics, molecular networking, untargeted, biomarker, metabolite

## Abstract

Ectopic pregnancy (EP) is the leading cause of maternal morbidity and mortality in the first trimester. Using an untargeted metabolomic approach, we sought to identify putative plasma biomarkers using tandem liquid chromatography–mass spectrometry for the detection of tubal EP. This case-control study included the prospective recruitment of 50 tubal EP cases and 50 early intrauterine pregnancy controls. To avoid over-fitting, logistic regression models were developed in a randomly selected discovery group (30 cases vs. 30 controls) and validated in the test group (20 cases vs. 20 controls). In total, 585 mass spectral features were detected, of which 221 molecular features were significantly altered in EP plasma (*p* < 0.05). Molecular networking and metabolite identification was employed using the Global Natural Products Social Molecular Networking (GNPS) database, which identified 97 metabolites at a high confidence level. Top significant metabolites include subclasses of sphingolipids, carnitines, glycerophosphocholines, and tryptophan metabolism. The top regression model, consisting of D-erythro-sphingosine and oleoyl-carnitine, was validated in a test group and achieved an area under receiving operating curve (AUC) (95% CI) = 0.962 (0.910–1) with a sensitivity of 100% and specificity of 95.9%. Metabolite alterations indicate alterations related to inflammation and abnormal placentation in EP. The validation of these metabolite biomarkers in the future could potentially result in improved early diagnosis.

## 1. Introduction

Ectopic pregnancy (EP) is the leading cause of maternal morbidity and mortality in the first trimester and accounts for 9% of all pregnancy-related deaths [1,2,3]. EP refers to embryo implantation at a location other than the endometrium of the uterine cavity. The most common ectopic location is the fallopian tubes [4]. The annual incidence of EP has been increasing over the past 30 years and is currently estimated at 20 cases per 1000 pregnancies [5]. Most patients with EP are initially asymptomatic or have nonspecific symptoms, including vaginal bleeding and low abdominal pain, which may be indistinguishable from intrauterine pregnancy (IUP), or threatened abortion. 

Current diagnostic evaluation of a suspected EP involves serial human chorionic gonadotropin (hCG) testing and pelvic ultrasounds [6]. However, an early diagnosis of EP remains challenging, as 40% of initial transvaginal ultrasound (TVUS) assessments for EP are inconclusive because most cases have not progressed to a stage where adnexal mass/pregnancy can be visualized [7,8]. Further, the sonographic visualization of an adnexal mass mostly remains suggestive but not diagnostic. Other adnexal pathologies, such as a paratubal cyst, corpus luteum cyst, hydrosalpinx, endometrioma, or bowel, could be mistaken for an EP [9]. Hence, the most common initial diagnosis will be a pregnancy of unknown location (PUL) until serial sonographic and laboratory evaluations can ultimately distinguish intrauterine pregnancy (IUP) vs. EP. An hCG level below the discriminatory threshold (3500 mIU/mL) is used to minimize a false positive diagnosis of EP that can result in the interruption of an IUP [10,11]. For these reasons, a delay in the accurate diagnosis of EP is common, and such delays increase the risks of tubal rupture and intraabdominal hemorrhage, which are significant causes of morbidity and mortality [12]. 

Despite the advancements in the management of EP, diagnostic tools have not improved in the last two to three decades, and there is still a lack of accurate biomarkers for the diagnosis of EP [13]. Several EP biomarkers related to inflammation and muscle damage [14], abnormal trophoblast function [15,16], angiogenesis [17], and impaired tubal transport [18,19] have been reported; however, none of them have as yet been validated for clinical use. Further, a proteomic approach has identified several serum proteins [20], including fibronectin [21], ADAM12 [22], isoforms of the beta-1 glycoprotein family, pregnancy-associated plasma protein A (PAPP-A), progestagen-associated endometrial protein, and chronic somatomammotropin precursor [23], that have the potential biomarker value, but none are currently being used in clinical practice [13,24]. 

Metabolomics characterize and quantify small molecule metabolites and their interactions within a biological system [25]. When compared to other omics sciences, metabolomics is said to provide the best characterization of the phenotype. For these reasons, metabolomics was recently characterized as the ‘stethoscope’ of the 21st century [26]. Metabolomics has led to the identification of biomarkers for and the understanding of the pathology of disorders related to women’s health [27,28,29]. Due to metabolites demonstrating a wide range of chemical properties, there is no single approach that can identify the whole metabolome. Targeted and untargeted metabolomic approaches have been utilized. We previously published the use of accurate targeted metabolomic biomarkers using Nuclear Magnetic Resonance metabolomics for the diagnosis of EP [30]. Untargeted metabolomics allows for the exploration of a wider range of chemical data compared to the targeted approach and discovery of previously unknown compounds. Recent advances in bioinformatics, including the development of the Global Natural Products Social Molecular Networking web platform (GNPS) have enabled a more comprehensive interpretation of untargeted metabolomic data and specific biochemical identification of compounds [31]. Given the promising results of our preliminary targeted work, we sought to identify further potential metabolite biomarkers for the early detection of EP by using an untargeted global metabolomic approach.

## 2. Results

In total, 50 tubal EP cases and 50 controls were biochemically profiled using LC-MS/MS. There were no significant differences between demographic and clinical risk factors except a previous EP and adnexal surgery when all cases were compared to controls (50 vs. 50) (Table S1). The mean gestational age for EP cases vs. IUP controls was at 6 weeks 5 days (±1 w 1 d) vs. 5 weeks 5 days (±2 w 1 d). The exact GA of 22 tubal EP cases could not be determined due to the nature of tubal pregnancy, as the last menstrual period is usually unreliable in EP due to irregular menstrual bleeding. However, all EP cases included herein were in the early first trimester, consistent with IUP controls, as they were contained within the fallopian tube and were not ruptured. Table 1 displays the comparison of demographic and clinical variables between the cases and controls in the discovery and validation set. Following the stratification of the samples into two groups, there were no significant differences found in age, body mass index (BMI), and parity between the two groups. However, a history of tubal and pelvic surgery and previous EP remained significant (*p* < 0.05) (Table 1). The finding of an adnexal mass on imaging that is suspicious of EP was found in 64% of EP cases. The mean β-hCG level in the cases was 2632 (SD: 481) mIU/mL vs. 3201 (1106) mIU/mL (*p* = 0.06) in the controls. 

**Figure 1:**
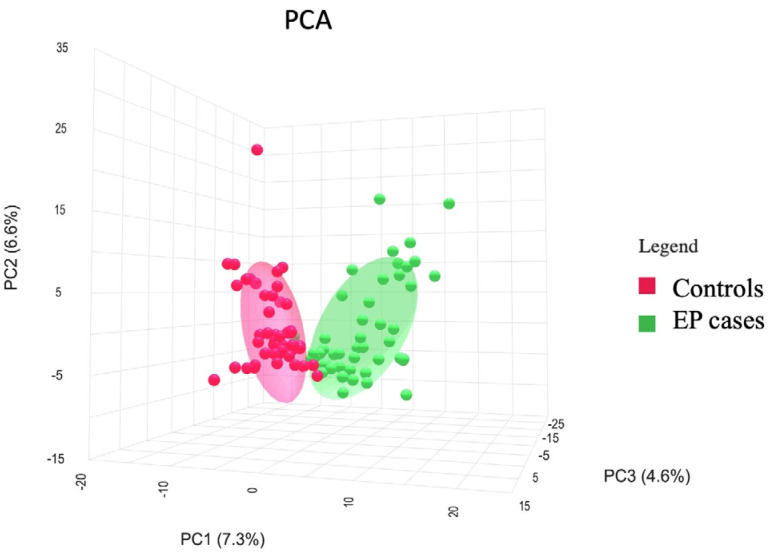
PCA plot showing controls (red) vs. EP cases (green).

Untargeted metabolomics identified a total of 585 molecular features (mass spectral features with unique MS/MS fragmentation patterns). Two hundred twenty-one molecular features were significantly altered in EP plasma (*p* < 0.05). Ninety-four features were increased, and one hundred twenty-seven features were decreased in cases, as presented in the volcano plot (Figure S1). The relative concentrations of each feature, their corresponding mass, and their statistical differences between EP and the controls are presented in Table S2. Using the 585 features, the PCA score plot showed great separation between the EP cases and controls (Figure 1). The PLS-DA plot findings are consistent with the PCA results, where a significant separation between the EP cases and controls is demonstrated (Figure S2). The separation on the PLS-DA was confirmed with permutation testing using 2000 repeats and the model achieved statistical significance (*p* = 0.0025). The heatmap using the top 50 molecular features was able to demonstrate good visual separation between the cases and controls (Figure S3).

Annotation could be retrieved, and chemical identification was determined for 97 features (mass spectrometry moieties) by matching them to GNPS libraries, where six mass spectrometry features were annotated at level 1, eight features at level 2, and the rest at level 3 [32]. Our annotation rate in this dataset was 16.5% (97 nodes out of 585). Out of 221 statistically significantly different molecular features in IUP vs. EP, those with unique identification are presented in Table 2 with their corresponding annotation level, raw formulas, feature identities, precursor masses, and *p*-values (<0.05). The top significantly altered metabolites included sphingolipids, glycerophosphocholines, carnitines, and members of the tryptophan metabolism pathway. These metabolites were all decreased in EP cases compared to the controls. Significantly altered sphingolipids included D-erythro-Sphingosine, sphingosine 1-phosphate, sphingomyelin (d18:1/24:0), N-Lauroyl-D-erythro-sphingosylphosphorylcholine, and N-Tetracosanoyl-4-sphingenyl-1-O-phosphorylcholine. Altered tryptophan metabolites included tryptophan betaine, phenylalanyltryptophan, and N-Methyl-L-tryptophan. Further, several glycerophosphocholines were dysregulated.

**Figure 2:**
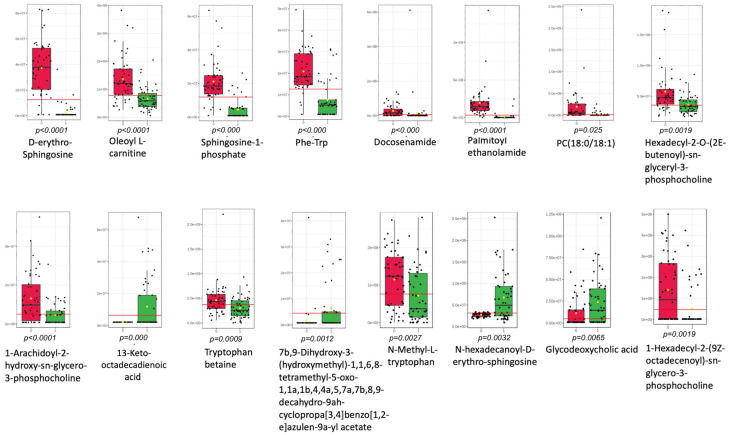
Boxplots for top significant molecular features (*p* < 0.05). 0: controls (red), 1: EP cases (green).

Figure 2 presents these metabolites with their corresponding boxplots when EP cases are compared to controls. Overall, the top 5 significant metabolites (*p* < 0.05) were D-erythro-Sphingosine, oleoyl L-carnitine, sphingosine 1-phosphate, phenylalanyl tryptophan, and docosenamide.

**Figure 3:**
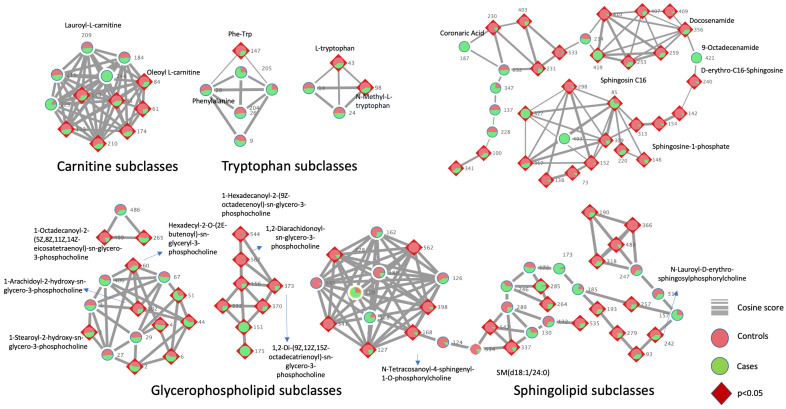
Comparative molecular networks between the EP cases (green) and controls (red). Nodes are represented as colored pie charts to indicate the distribution in the cases vs. controls. The diamond red border indicates the molecular features that are statistically significant between the EP cases vs. controls (*p* < 0.05).

The molecular network of the molecular structures identified using the GNPS libraries displays the members of sphingolipids, glycerophosphocholines, tryptophan, and carnitine metabolism, as presented in Figure 3. Individual ‘nodes’ represent a consensus MS/MS spectrum, and the ‘edges’ connecting the nodes demonstrate spectral similarity, which is determined by the cosine score. The pie chart inside the nodes shows the number of spectra found in the cases vs. controls. The red color in the pie charts indicates the controls, and green highlights the EP cases. To further ease the interpretation, we also identified the nodes with a diamond red border that indicated the significance (*p* < 0.05) when it was compared between the EP cases and controls. This analysis showed the potential of GNPS Molecular Networking in covering various chemical classes and the power of metabolome mining tools in expanding the interpretability of complex untargeted metabolomic data.

Regression models were generated in the discovery set (30 EP cases vs. 30 IUP controls) and achieved a predictive accuracy with AUC > 0.950, with a sensitivity and specificity that are presented in Table S3. The top diagnostic metabolite model (D-erythro-sphingosine + Oleoyl carnitine) in the discovery set achieved an AUC (95% CI) = 0.984 (0.940–1.000) with a sensitivity and a specificity of 100% and 96.8%, respectively. The regression models were tested using a separate validation set (20 EP cases vs. 20 IUP controls). A highly accurate predictive value was confirmed with an AUC (95% CI) = 0.962 (0.910–1), sensitivity of 100%, and specificity of 95.9% with a 10-fold CV. The second-best metabolite model (Palmitoyl ethanolamide + 9,10-cis epoxide of linoleic acid) achieved an AUC of 0.985 (0.965–1) with a sensitivity of 96% and specificity of 98% (Table 3). The integration of demographics into logistic models did not improve the accuracy, as only the metabolite model was highly accurate. Table S4 presents the logistic regression models that include the clinical predictors for ectopic pregnancy.

## 3. Discussion

In this untargeted metabolomic study, we report for the first time highly accurate diagnostic metabolite models for the detection of EP in a validation group. The metabolites that were altered include mostly lipids. Our top metabolite model, D-erythro-C18-Sphingosine and oleoyl L-carnitine, was highly predictive in a validation group, achieving an AUC = 0.962 (0.910–1.000) along with a sensitivity of 100% and specificity of 95.9%. The high sensitivity and specificity values were also reproduced in subsequent metabolite regression models with an AUC > 0.950. This demonstrates the strength of our predictive models, as different metabolite combinations can accurately predict EP cases. Our previous targeted metabolomic approach also highlighted the metabolite biomarkers that are capable of accurately detecting EP. In addition to the development of predictive biomarkers, we also demonstrated biochemical changes in EP cases with significant dysregulation in lipids, including altered sphingolipids, oleoyl-L-carnitine, and gylcerophosphocholines. Sphingolipids, including sphingosine-1-phosphate (S1P), and ceramides are involved in many biological signaling pathways. We found decreased levels of D-erythro-sphingosine, sphingosine 1-phosphate (S1P), sphingomyelin (d18:1/24:0), N-Lauroyl-D-erythro-sphingosylphosphorylcholine, and N-Tetracosanoyl-4-sphingenyl-1-O-phosphorylcholine in EP. Sphingosine and ceramide levels are known to increase with advancing gestation, suggesting a physiological role in parturition [33]. Further, S1P signaling is crucial for the function of corpus luteum, progesterone production, decidualization, and trophoblast invasion [34]. Impaired corpus luteum function and decreased progesterone production with abnormal decidualization is well known to occur in EP cases when compared to healthy IUPs [35]. Aberrant sphingolipid metabolism in human fallopian tubes has been demonstrated in EP. S1P concentration within the human oviduct from ectopic pregnancy was found to be reduced relative to normal pregnancy subjects [36]. These are consistent with our findings, where sphingolipids are decreased in EP cases and can potentially be used as a marker to differentiate from normal pregnancies. Further, oleoyl-L-carnitine was significantly decreased in EP cases, which is known to play an essential role in regulating the transportation of medium-chain and long-chain fatty acids across the inner mitochondrial membrane for β-oxidation. The plasma concentration of L-carnitine in pregnant women is reported to be much lower than that in nonpregnant women and continues to decline as gestation advances [37,38]. Further, carnitine levels were previously shown to be lower in patients who had spontaneous miscarriages when compared to healthy pregnancies [39]. L-carnitine also functions as a key metabolite to maintain the homeostasis between apoptosis and proliferation in trophoblast cells [40]. Compared to IUP, in tubal EP, there is abnormal trophoblastic growth and increased levels of apoptosis, and normal placentation does not occur [41,42]. Therefore, the decreased levels of L-carnitine found herein could potentially represent the disrupted progression of placentation in EP cases when compared to IUP controls. As part of our second and third regression models, palmitoylethanolamide and N-palmitoylethanolamine were shown to be good predictors of tubal EP. Our results indicate that these metabolites are decreased in EP cases when compared to controls. Palmitoylethanolamide is an endogenous fatty acid amide, which has an anti-inflammatory role through the inhibition of mast cells, monocytes, and macrophages [43]. This could represent the role of palmitoylethanolamide in healthy IUP, as early pregnancy is a state of anti-inflammation with elevated progesterone levels. EP is also known to have a more inflammatory response when compared to healthy IUP controls [21,44].

We also report decreased levels of several glycerophosphocholines in EP cases when compared to the controls. Glycerophosphorylcholine is a choline derivative and is formed in the breakdown of phosphatidylcholine. Phosphotidylcholines (PC) plays a major role in cell membranes and can be found in systemic circulation as bound to lipoproteins [45]. The cleavage of PC by phospholipase A2 produces inflammatory mediators, including lysophosphotidylcholines, which are the precursors for eicosanoids. Hence, the decreased levels of glycerophosphorylcholines could be due to an increased inflammatory process in EP cases that has been shown to have increased levels of IL-6, IL-8, TNF-α, and fibronectin [21,44]. Further, plasma choline levels are higher in serum during normal health pregnancies when compared to nonpregnant women [46].

In addition to the lipid dysregulation in EP cases, our results indicate a decrease in tryptophan metabolites, including phenylalanyltryptophan, tryptophan betaine, and N-methyl-L-tryptophan. Phenylalanyltryptophan also performed well diagnostically as part of our third regression model. Tryptophan is an indispensable amino acid, and higher levels of utilization are needed in pregnancy due to increased maternal and fetal protein synthesis. Additionally, tryptophan is converted to kynurenine by immune cells, which plays a crucial role in the regulation of immune responses during infections, inflammation, and pregnancy [47]. Although total serum tryptophan levels are decreased in healthy pregnancies, free tryptophan levels are increased. There is also an overexpression of the kynurenine pathway, which is highly expressed in the placenta and serum during pregnancy [48]. On the contrary, in EP cases, we show decreased levels of tryptophan metabolites when compared to IUP, which could be due to a pro-inflammatory state, the impaired implantation of the embryo, and abnormal placentation.

There are several strengths of this study. First, we utilized an untargeted MS-based metabolomic approach, which provides a high throughput and unbiased survey of chemical components in a biological sample. Despite these advantages, the use of untargeted metabolomics to develop biomarkers for human diseases has been historically challenging due to the technical difficulties of obtaining spectral annotation. Untargeted metabolomic output generates many spectra that correspond to thousands of unique molecules. In terms of chemical identification, currently, most of these spectra will not match existing spectral libraries. Hence, in our study, we utilized GNPS Molecular Networking to overcome this obstacle. GNPS allows for the identification of metabolites that are available in untargeted metabolomic dataset libraries [31]. Another strength of our study was the use of a separate discovery and a validation set for biomarker discovery. This is an important strategy for minimizing over-fitting when analyzing large volumes of data such as metabolomics. Further, we included only EP cases that were pathologically confirmed, avoiding a false positive diagnosis, which occurs in clinical practice. Tubal pregnancies are the most common form of EP cases and therefore clinically relevant for biomarker discovery. The validation of these highly accurate markers could potentially lead to the early diagnosis of EP and reduce the number of follow-up visits and delays in diagnosis, which will ultimately decrease the morbidity and mortality rates. Further, an earlier diagnosis of EP before patients become critically ill or with advanced gestation could allow for the option of medical treatment via methotrexate as opposed to surgical treatment, which is more morbid and more costly. Therefore, accurate metabolite markers could potentially lead to not only decreased morbidity and mortality but also reduced healthcare costs [49].

This study is not without limitations. First, we only included tubal EP cases and matched IUP controls in this cohort to generate metabolite markers. However, these promising markers could potentially be validated in cases with intrauterine non-viable pregnancies and ultimately rarer forms of ectopic pregnancies, such as ovarian and abdominal types. Second, despite the use of a cutting-edge tool, GNPS Molecular Networking, to identify metabolite markers, there remains a large group of unidentified compounds that is capable of distinguishing EP cases from IUP controls. With time, it is expected that with expanded source libraries, the number of unidentified features will decline significantly. Further efforts are needed to enhance our ability to identify individual spectra obtained by an untargeted metabolomic approach, which is an essential method for biomarker discovery. Lastly, our sample size was small to moderate; however, this was due to the exploratory nature of this investigation. We have attempted to mitigate the limitations of small sample sizes through rigorous statistical controls, including a discovery and validation set, and the application of robust analytical techniques. Nonetheless, we recognize that a larger independent validation cohort in future studies would strengthen the generalizability of the results.

In conclusion, we report for the first time an untargeted plasma metabolomic approach for the detection of EP. This approach appears to be highly accurate. The validation of these predictive models in larger and clinically diverse populations could lead to the early detection of EP and decreased morbidity and mortality. Further, we were able to elucidate the metabolomic pathogenesis of EP by using a novel molecular networking approach. A prominent feature appears to be the dysregulation of lipids in EP cases. The findings herein represent the promising role of metabolomics in the detection and understanding of tubal EP cases.

## 4. Materials and Methods

### 4.1. Study Population and Sample Collection

This was a prospective case-control cohort study that included the recruitment of cases and controls with the collection of clinical data and human samples. The recruitment and sample collection were performed at Zekai Tahir Burak (ZTB) Women’s Health, Education and Research Hospital, Ankara, Turkey, following Institutional Review Board approval (IRB # 2011-0914). Untargeted metabolomic analysis of the de-identified samples was performed at Michigan State University, East Lansing, MI, following an additional approval by the Institutional Review Board at Corewell Health, Royal Oak, MI (IRB# 2014-380). All procedures performed in this study followed the ethical standards of the institutional research committee, which are consistent with the 1964 Helsinki Declaration and its later amendments. The study protocol included the recruitment and prospective follow-up of patients with early IUP and patients with pregnancies of unknown locations who are suspected to have EP. All the participants provided informed written consent. Patient recruitment and selection of patients are presented as a flow chart in Figure S4.

Patients evaluated for possible EP and early IUP underwent TVUS performed by a clinician with extensive expertise in gynecologic ultrasound. Plasma samples were collected on the same day of the initial clinical and sonographic evaluation. The presence of an adnexal mass at the initial TVUS and quantitative hCG measurements were prospectively noted for suspected EP cases. An adnexal mass, suggestive of EP, had the ultrasound appearance of a heterogenous mass or extra uterine sac-like structure with or without identification of a yolk sac or embryo [50]. Following an initial evaluation, serial quantitative hCG measurements were obtained from those with pregnancy of unknown location if indicated. For the final analysis, however, only the patients (*n* = 50) with confirmed surgical/pathological diagnosis of EP with the presence of chorionic villi in the salpingectomy specimen were included to ensure accurate diagnosis. All EP cases were located in the fallopian tube, and non-tubal EP (cornual, ovarian, cervical) were excluded from the study. Additionally, to prevent any other confounding factors, hemodynamically unstable patients and those who had previously received methotrexate (MTX) treatment were excluded from the study.

Controls (*n* = 50) were chosen from age-matched IUP patients at the time of their early prenatal visit, and they met the criteria for control status if they subsequently delivered at term without any early pregnancy complications. IUP diagnosis was confirmed on the day of sample collection based on sonographic visualization of an intrauterine gestational sac with a yolk sac and/or an embryo, and non-viable pregnancies were excluded [50]. In total, 50 EP cases and 50 IUP controls were included in the analysis.

### 4.2. Sample Collection

Blood samples were collected at Zekai Tahir Burak Women’s Health, Education, and Research Hospital via venipuncture from each participant using tubes containing dipotassium EDTA. Blood specimens were expeditiously transferred to the laboratory, and whole blood centrifugation was initiated within 30 min following collection at 4000× *g* for 10 min at 4 °C. The plasma was collected, divided into 250 µL sized aliquots, and stored at −80 °C for analysis. All specimens were frozen within one hour of collection. Samples were thawed once and were utilized only for the metabolomic analyses presented herein.

### 4.3. Sample Extraction and LC-MS/MS Mass Spectrometry

All plasma samples were extracted by adding 100 µL of ice-cold 100% methanol to 100 µL of plasma. The extracts were mixed in a homogenizer at 700RPM for 10 min, sonicated at 4 °C for 20 min, and then centrifuged at 13,000× *g* at 4 °C for 20 min to pellet the protein. Extracts were stored at −80 °C prior to metabolomic analysis. The extracts were analyzed for untargeted metabolomic profiling using a Thermo QExactive^®^ mass spectrometer coupled to a Vanquish^®^ liquid chromatography system, MA, USA (LC-MS/MS) at the Michigan State University Research and Technology Support Facility in Lansing, MI. LC-MS/MS separation protocols and instrument methods were as described by Raghuvanshi et al. [51] Thermo .raw files were converted to .mzXML format for further metabolomic data analysis.

### 4.4. Feature-Based Molecular Networking Using GNPS and Compound Annotation

A molecular network was developed using the feature-based molecular networking workflow (https://ccms-ucsd.github.io/GNPSDocumentation/featurebasedmolecularnetworking/; accessed on 1 September 2023) on the GNPS website (http://gnps.ucsd.edu; accessed on 1 September 2023) by uploading the aggregated MS2 mass list [31]. The mass spectrometry data were first processed with MZMINE2 (cite accordingly, see below) and the results were exported to GNPS for feature-based molecular network analysis. The data were filtered by removing all MS/MS fragment ions within ±17 Da of the precursor *m*/*z*. MS/MS spectra were window-filtered by choosing only the top 6 fragment ions in the ±50 Da window throughout the spectrum. The precursor ion mass tolerance was set to 0.02 Da and the MS/MS fragment ion tolerance to 0.02 Da. A molecular network was then created where edges were filtered to have a cosine score above 0.7 and more than 4 matched peaks. Further, edges between two nodes were kept in the network if and only if each of the nodes appeared in each other’s respective top 10 most similar nodes. Finally, the maximum size of a molecular family was set to 100, and the lowest-scoring edges were removed from the network until the molecular family size was below this threshold. The spectra in the network were then searched against GNPS spectral libraries [52,53]. The library spectra were filtered in the same methodology as it was performed for the input data. All matches kept between network spectra and library spectra were required to have a score above 0.7 and at least 6 matched peaks. The molecular networks were visualized using Cytoscape 3.8.2. software [54].

### 4.5. Statistical Analysis

Demographic and clinical variables were compared between cases and controls and within the discovery and validation set. Student’s *t*-test or χ^2^ test were used based on the type of the variable. Metabolite concentrations that are normally distributed were compared using Student’s *t*-test, and non-normally distributed metabolites were compared using Mann–Whitney U test. The multivariate analysis was carried out using R 4.4.1. statistical software. All data were normalized to the sum and auto-scaled before Principal Component Analysis (PCA) and Partial Least Squares Discriminant Analysis (PLS-DA) [55]. Models were cross-validated using permutation testing (2000 iterations) to determine if the separation observed in PCA and PLS-DA plots was statistically significant. To validate the diagnostic models and minimize over-fitting, the entire dataset was randomly split into two groups, a discovery set (EP: 30, controls: 30) and a validation set (EP: 20, controls: 20). This method of splitting dataset into a discovery and validation set has been previously utilized in metabolomic studies [56,57,58]. Subsequent to this randomized allocation, there were no significant differences in demographics and any other potentially confounding variables between the two groups. Using the discovery group, logistic regression analysis was performed with a stepwise variable selection method, including LASSO (Least Absolute Shrinkage and Selection Operator) [59], to generate the diagnostic models. A k-fold cross-validation (CV) technique was employed to ensure the strength of the logistic regression models [60]. The average of the 10-fold CVs was used to determine the performance of the detection models developed using the discovery set. The diagnostic accuracy of the models obtained from the discovery set was also tested using the validation set, which included patients who were not used in deriving the model. To determine the performance of each regression model, the area under the receiver operating curve (AUROC or AUC), 95% confidence interval (CI), and sensitivity and specificity values were calculated for EP detection.

## Figures and Tables

**Table 1:** Demographic and clinical characteristics of ectopic pregnancy cases and control groups—discovery vs. validation/test group.

	Discovery Group			Validation Group		
Parameter	Cases	Controls	*p*-Value	Cases	Controls	*p*-Value
Number of patients	30	30		20	20	
Age, mean (SD)	25.7 (5.9)	26.1 (6.7)	0.147 ^	26.7 (5.2)	25.1 (5.7)	0.647 ^
Nullipara, *n*	13	16	0.343 *	18	22	0.248 *
BMI, mean (SD)	27.2 (5.3)	26.1 (5.9)	0.865 ^	25.2 (5.5)	25.1 (4.9)	0.955 ^
Risk Factors, *n*						
Previous EP	6	3	0.044 *	4	1	0.025 *
Previous Adnexal Surgery	7	3	0.046 *	5	2	0.038 *
Previous pelvic /abdominal Surgery	8	10	0.423 *	6	7	0.263 *
History of Infertility	3	4	0.520 *	1	4	0.608 *
History of PID	6	5	0.378 *	1	2	0.198 *
Smoking	7	5	0.102 *	8	9	0.092 *

^ Student *t*-test; * Chi-square.

**Table 2:** Top significant identified metabolite features between EP cases and controls (*p* < 0.05).

Compound Name	Annotation Level	Raw Formula	*m*/*z* [M + H]+	Feature ID	EP vs. IUP	*p*-Value
D-erythro-Sphingosine	1	C_18_H_37_NO_2_	300.289	240	Down	<0.0001
Sphingosine 1-phosphate	1	C_18_H_38_NO_5_P	380.255	146	Down	<0.0001
Oleoyl L-carnitine	1	C_25_H_47_NO_4_	426.357	84	Down	<0.0001
Phe-Trp	2	C_20_H_21_N_3_O_3_	352.165	147	Down	<0.0001
Docosenamide	2	C_22_H_43_NO	338.341	356	Down	<0.0001
Hexadecyl-2-O-(2E-butenoyl)-sn-glyceryl-3-phosphocholine	3	C_28_H_56_NO_7_P	550.386	60	Down	<0.0001
1-Arachidoyl-2-hydroxy-sn-glycero-3-phosphocholine	3	C_28_H_58_NO_7_P	552.402	102	Down	<0.0001
Palmitoyl ethanolamide	3	C_18_H_37_NO_2_	300.289	73	Down	<0.0001
1,2-Di-(9Z,12Z,15Z-octadecatrienoyl)-sn-glycero-3-phosphocholine	2	PC(18:3(9Z,12Z,15Z)/ 18:3(9Z,12Z,15Z))	778.538	373	Down	0.0001
13-ketooctadecadienoic acid	3	C_18_H_30_O_3_	295.226	89	Up	0.0001
N-Tetracosanoyl-4-sphingenyl-1-O-phosphorylcholine	3	C_41_H_83_N_2_O_6_P	815.697	328	Down	0.0003
Tryptophan betaine	3	C_14_H_18_N_2_O_2_	247.143	43	Down	0.0009
7b,9-Dihydroxy-3-(hydroxymethyl)-1,1,6,8-tetramethyl-5-oxo-1,1a,1b,4,4a,5,7a,7b,8,9-decahydro-9ah-cyclopropa[3,4]benzo[1,2-e]azulen-9a-yl acetate	1	C_22_H_30_O_6_	432.238	432	Up	0.0012
1-Hexadecyl-2-(9Z-octadecenoyl)-sn-glycero-3-phosphocholine	3	C_42_H_84_NO_7_P	746.606	544	Down	0.0019
N-Methyl-L-tryptophan	3	C_12_H_14_N_2_O_2_	188.070	98	Down	0.0027
N-hexadecanoyl-D-erythro-sphingosine	1	C_34_H_67_NO_3_	274.274	85	Down	0.0032
Glycodeoxycholic acid	1	C_26_H_43_NO_5_	450.321	41	Up	0.0065
1-Stearoyl-2-hydroxy-sn-glycero-3-phosphocholine	2	C_26_H_54_NO_7_P	524.371	3	Down	0.0158
Sphingomyelin (d18:1/24:0)	2	SM (d18:1/24:0)	814.688	542	Down	0.0199
1-Octadecanoyl-2-(5Z,8Z,11Z,14Z-eicosatetraenoyl)-sn-glycero-3-phosphocholine	3	PC (18:0/18:1)	810.597	488	Down	0.0251
N-Lauroyl-D-erythro-sphingosylphosphorylcholine	2	12:0 SM (d18:1/12:0)	647.512	242	Down	0.0259

**Table 3:** The performance of regression models in the validation group following a 10-fold cross-validation (validation group).

Metabolite Algorithms	AUC (95% CI)	Sensitivity	Specificity
D-erythro-C18-Sphingosine + Oleoyl L-carnitine	0.962 (0.910–1.000)	100%	95.9%
Palmitoyl ethanolamide + D-erythro-C18-Sphingosine	0.963 (0.914–1.000)	98.0%	94.0%
N-Palmitoylethanolamine + D-erythro-C18-Sphingosine + Sphingosine 1-phosphate + Phenylalanyl tryptophan	0.955 (0.902–1.000)	92.0%	98.0%

## Data Availability

Data are contained within the article or Supplementary Materials.

## Supplementary Materials

The following supporting information can be downloaded at https://www.mdpi.com/article/10.3390/ijms251910333/s1.
