# Supplementary material for: Untargeted Metabolomic Biomarker Discovery for the Detection of Ectopic Pregnancy
Source: Int J Mol Sci. 2024 Sep 26;25(19):10333. doi: 10.3390/ijms251910333 (PMC11476625; doi:10.3390/ijms251910333)
Supplement: Supplementary file 1 [file ijms-25-10333-s001.zip › ijms-3205794-supplementary.pdf]

Supplementary Material

Untargeted Metabolomic Biomarker discovery for the detection of Ectopic Pregnancy

Supplementary Figure S1. Volcano plot presenting molecular features when EP cases compared to controls (p-value <0.05)

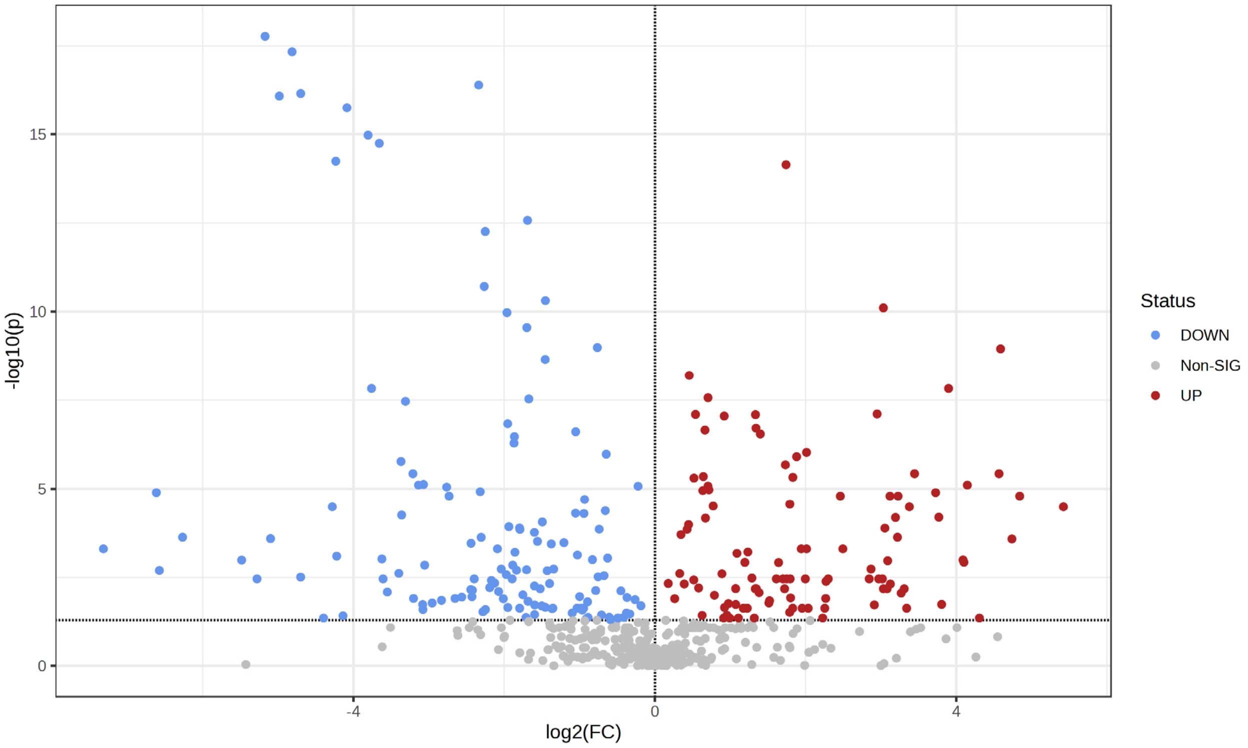

Blue indicating down regulated features (*n*=94), red indicated upregulated features (*n*=127)

Supplementary Figure S2. PLS-DA plot (EP cases vs IUP controls)

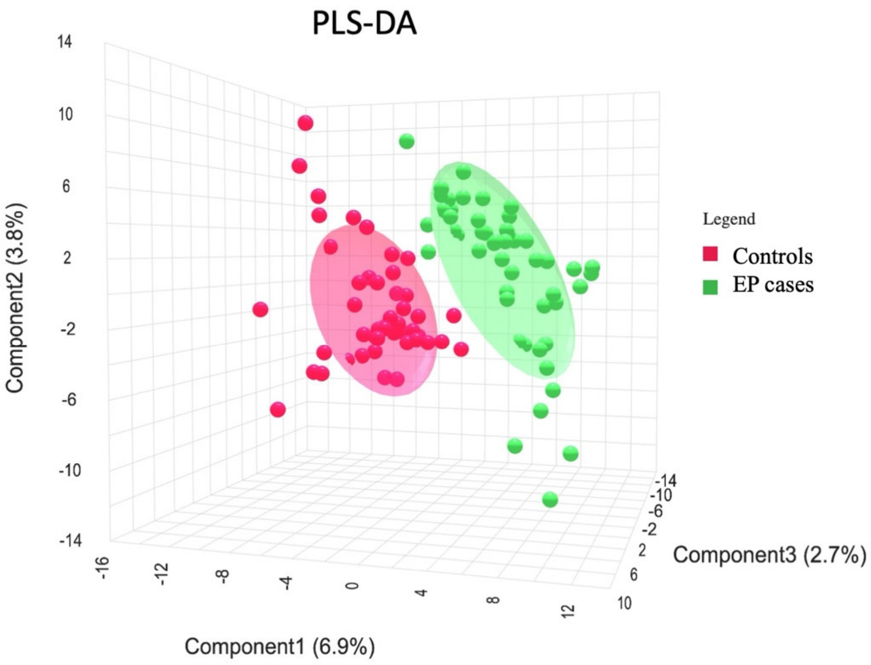

Supplementary Figure S3. Heatmap using top 50 mass spectrometry features between EP cases and controls

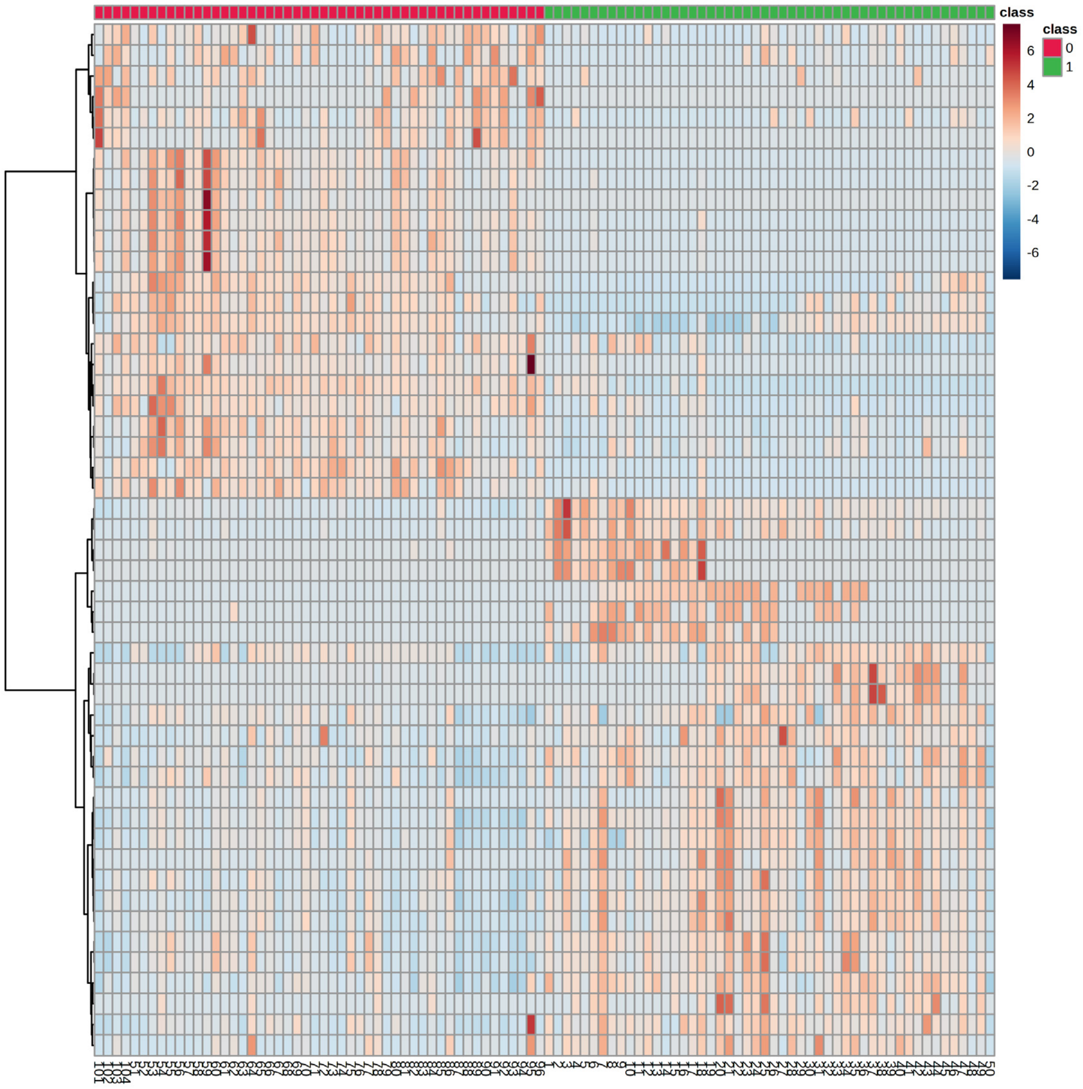

Top 50 mass spectrometry features in rows, red indicate controls, green indicate EP cases

Supplementary Figure S4. Study Population

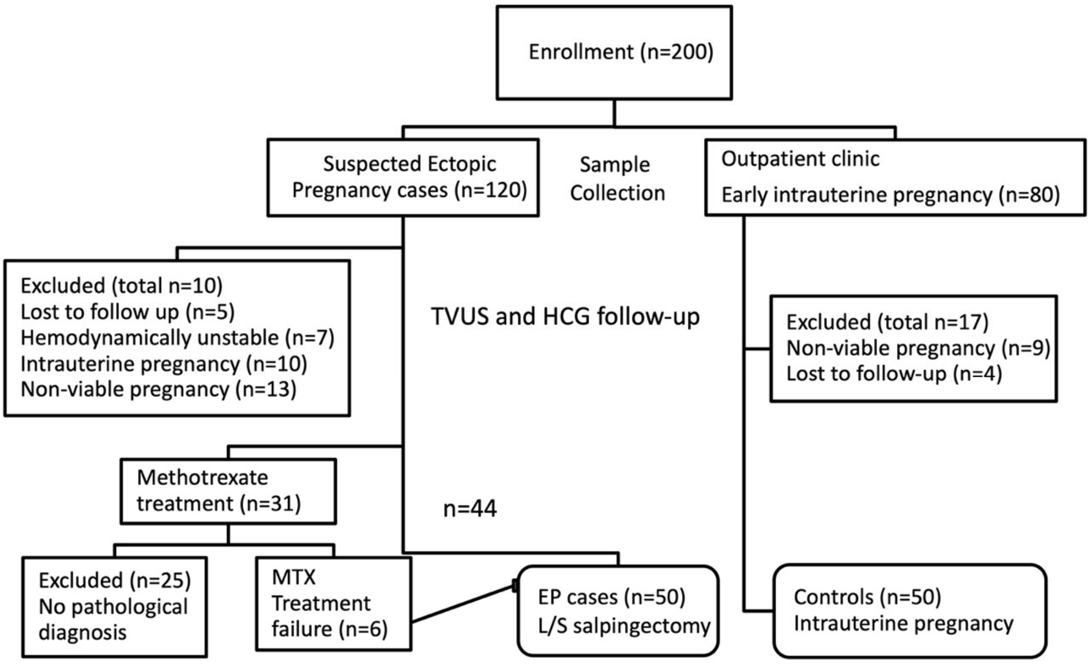

Supplementary Table S1. Demographic and clinical characteristics of ectopic pregnancy cases and control groups (all EP cases vs all IUP controls)

**Parameter**	Cases	Controls	*p*- value
Number of patients	50	50	
Age, mean (SD)	26.5 (5.9)	25.8 (6.8)	0.096^
Nullipara, n	31	38	0.077*
BMI, mean (SD)	26.2 (6.5)	25.9 (5.8)	0.180^
Risk Factors, *n*			
Previous EP	10	4	**0.022***
Previous Adnexal Surgery	12	5	**0.035***
Previous pelvic /abdominal Surgery	14	17	0.645*
History of Infertility	4	8	0.460*
History of PID	7	7	0.890*
Smoking	15	14	0.750*

.

Supplementary Table S2. Univariate analysis of molecular features when EP cases were compared to controls

Cluster Index_Precursor MZ	Mean (SD) of 0	Mean (SD) of 1	Controls vs EP	Fold change	*p*-value
10_991.672	875957367.062 (221731050.172)	516162869.638 (259269499.880)	Down	-1.7	**< 0.0001 (W)**
113_678.470	1620009.964 (2187193.254)	13262091.109 (12841156.037)	Up	8.19	**< 0.0001 (W)**
138_328.320	114623973.887 (106496208.425)	4073741.827 (16085668.546)	Down	-28.14	**< 0.0001 (W)**
142_371.326	42100026.463 (38770571.263)	1620089.687 (3593040.432)	Down	-25.99	**< 0.0001 (W)**
146_380.255	21208360.025 (12496039.243)	4455874.820 (6326413.954)	Down	-4.76	**< 0.0001 (W)**
147_352.165	20865282.497 (9673594.919)	7610933.667 (8600429.942)	Down	-2.74	**< 0.0001 (W)**
152_326.305	23467442.067 (21833436.893)	1247066.866 (1852628.597)	Down	-18.82	**< 0.0001 (W)**
154_399.358	47299037.803 (45806061.174)	1495097.088 (2568441.382)	Down	-31.64	**< 0.0001 (W)**
156_754.538	29047327.961 (24454662.813)	7503556.610 (9520270.003)	Down	-3.87	**< 0.0001 (W)**
17_355.0634	114169817.455 (59800186.763)	420704247.553 (380279891.378)	Up	3.68	**< 0.0001 (W)**
18_654.331	111213302.633 (148042175.677)	173721776.976 (127864377.305)	Up	1.56	**< 0.0001 (W)**
183_415.211	2964895.636 (8121609.781)	10553195.721 (13095618.740)	Up	3.56	**< 0.0001 (W)**
2_520.339	3113911963.200 (734903112.485)	4272826287.180 (1093301581.957)	Up	1.37	**< 0.0001 (W)**
21_478.293	86137245.489 (32441619.171)	140472500.631 (49492772.182)	Up	1.63	**< 0.0001**
240_300.289	37191447.285 (21116562.645)	2949063.001 (5813508.712)	Down	-12.61	**< 0.0001 (W)**
249_812.616	2598086349.208 (2164089918.448)	711325443.488 (1054285908.532)	Down	-3.65	**< 0.0001 (W)**
25_502.293	72728123.698 (21172891.953)	105729975.669 (30310993.307)	Up	1.45	**< 0.0001**
251_788.606	195441386.739 (390320375.725)	18921250.681 (46118119.540)	Down	-10.33	**< 0.0001 (W)**
298_354.336	51056146.127 (42433856.184)	1415551.853 (0.000)	Down	-36.07	**< 0.0001**
30_701.559	773377296.862 (1270400381.511)	282610164.125 (175397293.929)	Down	-2.74	**< 0.0001 (W)**
309_288.289	14708804.679 (15184666.460)	3774018.287 (12248123.621)	Down	-3.9	**< 0.0001 (W)**
32_159.112	404601503.830 (133485919.848)	80042356.271 (94747309.897)	Down	-5.05	**< 0.0001 (W)**
349_568.427	32320680.300 (41641918.510)	8880028.184 (12952166.379)	Down	-3.64	**< 0.0001 (W)**
356_338.341	28296072.912 (33869911.205)	18091535.734 (87809560.292)	Down	-1.56	**< 0.0001 (W)**
384_1487.003	11920286.611 (6323575.303)	3671976.686 (4579825.805)	Down	-3.25	**< 0.0001 (W)**
39_354.284	354117109.788 (238930673.164)	25319687.205 (75349406.307)	Down	-13.99	**< 0.0001 (W)**
40_526.292	52904345.054 (20076239.092)	83900192.990 (31357100.594)	Up	1.59	**< 0.0001**
44_570.353	14710354.106 (15928847.985)	27843710.577 (13268059.104)	Up	1.89	**< 0.0001 (W)**
51_518.324	12475268.492 (19886113.017)	31632747.854 (23390463.475)	Up	2.54	**< 0.0001 (W)**
58_478.293	6843030.989 (6253379.958)	18056904.200 (11797770.627)	Up	2.64	**< 0.0001 (W)**
6_546.355	281180896.152 (129725757.322)	402952192.116 (151322337.161)	Up	1.43	**< 0.0001 (W)**
61_424.341	166442793.098 (92111428.532)	51607178.150 (27031831.612)	Down	-3.23	**< 0.0001 (W)**
64_675.543	387752716.468 (683470676.311)	121668588.932 (125433170.207)	Down	-3.19	**< 0.0001 (W)**
72_1043.703	109449393.631 (70058990.152)	22788716.744 (44817794.411)	Down	-4.8	**< 0.0001 (W)**
73_300.289	196543094.817 (217278515.007)	11572046.680 (41114521.138)	Down	-16.98	**< 0.0001 (W)**
77_542.324	11628700.792 (15543704.673)	29351417.146 (20650949.146)	Up	2.52	**< 0.0001 (W)**
78_650.439	16221400.324 (9581667.173)	54223736.499 (37120554.019)	Up	3.34	**< 0.0001 (W)**
79_584.397	8971340.444 (0.000)	215800837.993 (211005849.970)	Up	24.05	**< 0.0001**
84_426.357	136614754.070 (79497002.296)	65968792.024 (40674128.244)	Down	-2.07	**< 0.0001 (W)**
88_997.625	1514449.207 (2136562.332)	6112796.401 (6200465.896)	Up	4.04	**< 0.0001 (W)**
90_1021.625	1192117.858 (1138055.254)	3961521.606 (3925091.336)	Up	3.32	**< 0.0001 (W)**
91_1257.827	993198.849 (0.000)	7674064.623 (8553179.019)	Up	7.73	**< 0.0001**
11_1039.672	176386558.439 (74437539.416)	274185150.067 (125783598.873)	Up	1.55	**< 0.0001 (W)**
252_811.669	524888695.984 (888729738.169)	105300655.720 (147670377.706)	Down	-4.98	**< 0.0001 (W)**
264_787.668	509023364.904 (585864249.933)	75016033.981 (232719103.332)	Down	-6.79	**< 0.0001 (W)**
294_838.632	152951028.084 (196803341.160)	15429488.828 (0.000)	Down	-9.91	**< 0.0001**
31_637.305	32992572.801 (57335388.397)	53786847.288 (46925336.398)	Up	1.63	**< 0.0001 (W)**
317_288.289	6173032.404 (6398620.854)	5290337.128 (18532682.397)	Down	-1.17	**< 0.0001 (W)**
366_780.553	78540954.195 (521465596.987)	801116.375 (419610.979)	Down	-98.04	**< 0.0001 (W)**
409_296.258	16671153.322 (30609459.325)	1984616.121 (5135705.964)	Down	-8.4	**< 0.0001 (W)**
427_318.300	944227.944 (0.000)	14078330.157 (18569365.809)	Up	14.91	**< 0.0001**
45_546.355	24791743.778 (16958853.804)	40774244.358 (20572407.301)	Up	1.64	**< 0.0001 (W)**
66_798.564	3257495.866 (1706220.309)	43106896.698 (74314908.262)	Up	13.23	**< 0.0001 (W)**
93_673.528	44812516.270 (26607406.881)	23478653.663 (17242530.890)	Down	-1.91	**< 0.0001 (W)**
35_480.308	33351870.258 (17838401.320)	57037290.571 (30209469.480)	Up	1.71	**< 0.0001 (W)**
56_1039.672	3707912.263 (5165285.448)	12836061.478 (13252749.292)	Up	3.46	**< 0.0001 (W)**
313_310.310	10868211.279 (15880697.806)	802418.880 (0.000)	Down	-13.54	**< 0.0001**
60_550.386	57572418.987 (36391404.722)	36504363.941 (15463122.121)	Down	-1.58	**< 0.0001 (W)**
149_314.232	34957848.909 (23104682.194)	18203449.879 (23035521.763)	Down	-1.92	**< 0.0001 (W)**
253_280.263	49822789.893 (81950571.554)	24025366.991 (58986893.545)	Down	-2.07	**< 0.0001 (W)**
304_720.590	63531602.341 (89553510.363)	6862244.762 (0.000)	Down	-9.26	**< 0.0001**
8_568.339	260326403.845 (100011810.889)	355037418.904 (121827515.276)	Up	1.36	**< 0.0001**
123_772.549	997009.663 (1210352.062)	13590970.731 (25741867.689)	Up	13.63	**< 0.0001 (W)**
318_800.619	13156700.173 (24992228.921)	1281228.865 (3298611.156)	Down	-10.27	**< 0.0001 (W)**
33_523.358	37400055.836 (27160894.407)	59571548.746 (28831264.820)	Up	1.59	**< 0.0001 (W)**
102_552.402	13094408.592 (12593182.599)	4651012.725 (5845971.637)	Down	-2.82	**< 0.0001 (W)**
370_804.553	25196489.156 (25991620.827)	6579411.198 (11326353.761)	Down	-3.83	**0.0001 (W)**
373_778.538	18821582.408 (20928661.841)	5421740.212 (8756129.665)	Down	-3.47	**0.0001 (W)**

100_547.436	16320788.358 (12003556.814)	9782416.499 (14361230.204)	Down	-1.67	**0.0001 (W)**
133_520.339	584405114.007 (218622335.453)	786526939.697 (331581305.759)	Up	1.35	**0.0001 (W)**
293_788.607	21496196.034 (26014260.380)	6213233.514 (10893573.886)	Down	-3.46	**0.0001 (W)**
89_295.226	2143130.702 (0.000)	11805446.052 (16506734.855)	Up	5.51	**0.0001**
560_1001.692	2917378.926 (4091698.757)	963293.555 (693084.817)	Down	-3.03	**0.0002 (W)**
254_616.176	85462753.298 (88974370.320)	108700869.569 (338652485.731)	Up	1.27	**0.0002 (W)**
575_798.565	7702586.327 (11512950.421)	1160392.631 (0.000)	Down	-6.64	**0.0002**
114_826.595	1026617.382 (537724.528)	9566044.293 (17336219.990)	Up	9.32	**0.0002 (W)**
279_759.637	93800110.098 (172145773.003)	1217546.933 (637730.143)	Down	-77.04	**0.0002 (W)**
112_536.334	890161.473 (0.000)	9704169.706 (15746959.979)	Up	10.9	**0.0002**
408_296.258	12346391.070 (23591152.702)	2498755.183 (7346399.545)	Down	-4.94	**0.0002 (W)**
310_770.608	28572691.375 (59127877.196)	833081.262 (557588.716)	Down	-34.3	**0.0003 (W)**
96_790.558	2714468.738 (4491986.151)	72475960.948 (171439913.191)	Up	26.7	**0.0003 (W)**
71_814.559	4392997.184 (0.000)	104164133.537 (180341828.144)	Up	23.71	**0.0003**
151_756.553	852673.568 (0.000)	7415772.609 (11905654.661)	Up	8.7	**0.0003**
407_298.274	19398070.098 (40286337.072)	6590482.577 (34175654.241)	Down	-2.94	**0.0003 (W)**
177_842.590	895005.523 (0.000)	9304409.596 (15387029.358)	Up	10.4	**0.0003**
95_538.386	14054909.552 (15567494.526)	6089661.335 (6189316.513)	Down	-2.31	**0.0003 (W)**
328_815.697	7992493.613 (14132223.395)	1473578.126 (4175248.361)	Down	-5.42	**0.0003 (W)**
231_595.493	41392521.575 (61487245.119)	15966886.209 (47244049.030)	Down	-2.59	**0.0004 (W)**
567_830.570	16537034.915 (29746667.143)	851047.410 (0.000)	Down	-19.43	**0.0005**
175_756.553	959527.458 (0.000)	8777898.764 (14932965.599)	Up	9.15	**0.0005**
111_1063.674	1204219.141 (1051554.930)	2833360.512 (3271829.892)	Up	2.35	**0.0006 (W)**
121_838.558	3043292.780 (0.000)	53921031.221 (98361849.724)	Up	17.72	**0.0006**
492_568.427	5684553.185 (7578860.540)	1569424.430 (2656896.369)	Down	-3.62	**0.0006 (W)**
42_812.615	348125993.857 (659738670.908)	740905184.914 (872860800.346)	Up	2.13	**0.0007 (W)**
259_280.263	20121246.147 (31032727.553)	9870575.318 (23675897.261)	Down	-2.04	**0.0007 (W)**
303_774.602	46880514.078 (115242116.903)	2511587.175 (4219687.561)	Down	-18.67	**0.0008 (W)**
43_247.143	462931876.346 (313458193.638)	299838361.354 (206048655.863)	Down	-1.54	**0.0009 (W)**
69_584.397	874897.414 (0.000)	25088392.637 (48417522.545)	Up	28.68	**0.0009**
220_284.294	7681704.657 (10469537.885)	4326397.918 (12796017.284)	Down	-1.78	**0.0010 (W)**
140_790.558	1316946.392 (3221387.908)	22424407.881 (53178887.142)	Up	17.03	**0.0010 (W)**
305_772.621	46526173.103 (92868368.378)	1039837.225 (861831.232)	Down	-44.74	**0.0010 (W)**
141_594.376	1394983.421 (1992772.238)	11878858.582 (26215719.226)	Up	8.52	**0.0011 (W)**
54_802.596	4418711.651 (12520777.036)	75878019.159 (117671055.768)	Up	17.17	**0.0012 (W)**
119_808.581	7832494.638 (32672661.852)	17928853.401 (38256470.745)	Up	2.29	**0.0012 (W)**
432_432.238	1859353.083 (5784029.268)	5803107.741 (9350575.745)	Up	3.12	**0.0012 (W)**
296_746.606	119826911.905 (244966522.310)	750965.338 (0.000)	Down	-159.56	**0.0012**
284_1309.964	9266405.903 (16261830.497)	1115190.610 (771017.997)	Down	-8.31	**0.0014 (W)**
300_785.653	4647864.857 (6878834.151)	1257359.677 (912546.383)	Down	-3.7	**0.0014 (W)**
544_760.585	3097249.213 (5194123.508)	951659.752 (502578.841)	Down	-3.25	**0.0019 (W)**
403_612.520	57798999.317 (88823817.461)	16192128.687 (36066177.504)	Down	-3.57	**0.0020 (W)**
295_785.653	139761794.785 (318112697.379)	1465794.156 (4117090.440)	Down	-95.35	**0.0020 (W)**
190_746.606	140770287.079 (157081719.932)	52293906.617 (102154532.373)	Down	-2.69	**0.0020 (W)**
148_622.408	829896.252 (0.000)	7774759.548 (15346308.581)	Up	9.37	**0.0024**
410_280.263	15009008.536 (36016702.554)	1424337.143 (2091767.365)	Down	-10.54	**0.0024 (W)**
28_1063.672	69389723.007 (25961555.051)	87241285.958 (37151017.602)	Up	1.26	**0.0024 (W)**
94_518.324	8499557.601 (9868203.354)	15755258.824 (12707136.720)	Up	1.85	**0.0025 (W)**
258_787.603	97738186.105 (139950959.631)	24925445.495 (45354306.675)	Down	-3.92	**0.0026 (W)**
98_188.070	115145988.100 (75720682.326)	72170476.847 (71216031.982)	Down	-1.6	**0.0028 (W)**
564_528.245	7145658.365 (14305883.810)	812706.121 (0.000)	Down	-8.79	**0.0029**
99_585.270	36937162.461 (28372157.090)	21920966.609 (23142245.809)	Down	-1.69	**0.0030 (W)**
262_796.621	168770039.842 (311306128.405)	6491752.226 (33315406.020)	Down	-26	**0.0031 (W)**
85_274.274	27598710.590 (4716664.733)	67398207.215 (55733139.914)	Up	2.44	**0.0033 (W)**
178_826.594	813011.945 (0.000)	3281908.383 (5655937.907)	Up	4.04	**0.0033**
256_798.565	12084462.574 (32701346.849)	17285858.241 (93194948.634)	Up	1.43	**0.0037 (W)**
322_872.770	28471383.821 (83778331.847)	6331356.880 (33699450.104)	Down	-4.5	**0.0038 (W)**
180_584.398	22200322.007 (80553465.791)	107216270.355 (191746978.632)	Up	4.83	**0.0041 (W)**
490_872.769	36453628.754 (141539515.980)	8350801.969 (47936178.464)	Down	-4.37	**0.0045 (W)**
4_522.355	2858039142.900 (999614841.637)	3232832477.120 (654056149.174)	Up	1.13	**0.0047 (W)**
332_822.636	7451428.942 (17725166.212)	2828981.705 (6640562.889)	Down	-2.63	**0.0047 (W)**
334_675.543	3372165.771 (17358770.744)	29435624.061 (64602782.839)	Up	8.73	**0.0048 (W)**
570_112.087	3607798.379 (6685900.459)	848140.416 (0.000)	Down	-4.25	**0.0053**
528_111.091	3102530.660 (4641357.879)	1025234.369 (701778.949)	Down	-3.03	**0.0055 (W)**
53_454.292	23883609.131 (12543159.813)	31299330.702 (13608626.475)	Up	1.31	**0.0056**
105_796.547	892697.665 (0.000)	7407892.516 (15897737.173)	Up	8.3	**0.0056**
230_593.476	10213714.345 (35450206.221)	2236773.220 (2984015.339)	Down	-4.57	**0.0061 (W)**
49_572.371	16823759.793 (13474730.883)	25167962.365 (16290982.072)	Up	1.5	**0.0062 (W)**
192_772.549	887497.306 (0.000)	6483418.415 (13876866.369)	Up	7.31	**0.0064**
41_450.321	11346059.674 (18315207.027)	23857609.035 (27102507.334)	Up	2.1	**0.0065 (W)**
287_808.582	5646714.308 (13624837.008)	1038710.900 (544058.905)	Down	-5.44	**0.0069 (W)**
417_225.195	16958032.912 (0.000)	51848321.621 (87786725.060)	Up	3.06	**0.0071**
281_744.550	14796756.943 (31381342.904)	2762680.959 (1760462.120)	Down	-5.36	**0.0072 (W)**
165_746.532	870425.760 (0.000)	4907238.521 (10182110.525)	Up	5.64	**0.0072**

468_1017.688	16491223.741 (12981753.359)	9570481.697 (10279823.115)	Down	-1.72	**0.0073 (W)**
160_636.423	1035748.751 (0.000)	3982933.660 (7456545.188)	Up	3.85	**0.0074**
22_544.339	162797761.562 (57756364.780)	195384120.906 (61465447.853)	Up	1.2	**0.0075**
174_368.279	28305935.571 (24418222.117)	20715397.782 (27880113.220)	Down	-1.37	**0.0075 (W)**
398_815.697	6361236.667 (15037711.037)	1511297.072 (4037109.337)	Down	-4.21	**0.0078 (W)**
541_815.698	44512538.805 (168951787.674)	3799775.390 (8476382.418)	Down	-11.71	**0.0081 (W)**
429_1102.310	1082501.948 (1350722.286)	2824076.507 (5238634.531)	Up	2.61	**0.0084 (W)**
144_830.553	908742.618 (0.000)	38970678.173 (98016698.821)	Up	42.88	**0.0084**
75_822.563	7519423.358 (19798101.842)	72484064.592 (119468649.575)	Up	9.64	**0.0087 (W)**
581_112.087	3729939.295 (7132881.471)	1003660.135 (0.000)	Down	-3.72	**0.0094**
415_211.180	24247932.620 (0.000)	78814291.019 (142968436.425)	Up	3.25	**0.0095**
379_111.091	13685224.864 (25815942.545)	4065015.270 (8192453.541)	Down	-3.37	**0.0097 (W)**
164_796.621	26202410.119 (78154359.113)	45321607.932 (86097581.043)	Up	1.73	**0.0100 (W)**
307_701.559	9303358.778 (10647296.577)	4658107.959 (8598984.691)	Down	-2	**0.0108 (W)**
543_112.087	7331517.656 (14980695.167)	1363395.120 (2518238.851)	Down	-5.38	**0.0110 (W)**
360_872.769	44096791.071 (177620418.024)	7466461.875 (45725657.203)	Down	-5.91	**0.0112 (W)**
301_744.594	38612096.799 (95279534.820)	3135465.686 (0.000)	Down	-12.31	**0.0113**
83_400.342	57617578.573 (27454095.981)	44607952.083 (19742970.454)	Down	-1.29	**0.0115 (W)**
437_798.565	8206981.353 (24601243.810)	28603903.400 (139265039.337)	Up	3.49	**0.0118 (W)**
150_806.554	4696015.390 (0.000)	16304528.397 (31444664.401)	Up	3.47	**0.012**
562_815.697	3293908.401 (6761242.721)	801958.864 (0.000)	Down	-4.11	**0.0121**
414_227.156	25542259.840 (0.000)	101967808.204 (207523590.269)	Up	3.99	**0.0122**
573_111.091	1915349.572 (3154185.643)	755067.425 (0.000)	Down	-2.54	**0.0123**
319_111.091	8348307.674 (13226859.654)	2071035.260 (3722693.035)	Down	-4.03	**0.0125 (W)**
139_316.248	61068826.200 (36124022.668)	50912559.246 (58347769.902)	Down	-1.2	**0.0133 (W)**
163_814.559	877749.235 (0.000)	6306672.916 (15035566.867)	Up	7.19	**0.0138**
168_815.697	19914897.549 (63196925.285)	2797749.208 (11947850.683)	Down	-7.12	**0.0138 (W)**
117_788.542	1098434.126 (1228146.825)	3155145.009 (6205903.520)	Up	2.87	**0.0142 (W)**
118_814.559	1209108.615 (0.000)	4066508.213 (7958554.792)	Up	3.36	**0.0144**
127_815.697	10524479.311 (22821739.417)	3277495.218 (11188745.921)	Down	-3.21	**0.0147 (W)**
302_811.668	8262078.772 (9059006.212)	4448047.670 (6436579.145)	Down	-1.86	**0.0152 (W)**
3_524.371	4239358547.340 (1134435785.560)	3730107401.800 (929345121.318)	Down	-1.14	**0.0158**
226_561.412	1483312.847 (4079313.353)	4235694.241 (15184385.399)	Up	2.86	**0.0164 (W)**
265_752.559	33465551.433 (79775269.367)	4315340.020 (20253345.632)	Down	-7.76	**0.0165 (W)**
219_840.572	2233041.784 (0.000)	5680193.722 (9835490.960)	Up	2.54	**0.0167**
48_432.3108	11877446.503 (19036153.972)	23360599.952 (27412591.314)	Up	1.97	**0.0171 (W)**
285_787.667	2705423.891 (8090997.366)	37841221.903 (116224167.996)	Up	13.99	**0.0180 (W)**
426_1102.185	2819177.098 (6752488.757)	5932924.415 (9826355.251)	Up	2.1	**0.0181 (W)**
533_612.520	11097545.137 (34503872.438)	1311161.076 (1501559.559)	Down	-8.46	**0.0183 (W)**
86_774.564	3657909.238 (0.000)	9216620.737 (16105989.528)	Up	2.52	**0.0183**
87_942.467	1986832.850 (2545840.908)	14962690.219 (22096125.544)	Up	7.53	**0.0186 (W)**
257_759.637	53055370.991 (74598197.353)	17567809.696 (37083846.566)	Down	-3.02	**0.0187 (W)**
337_814.688	2876343.076 (6003752.359)	1016360.459 (701719.646)	Down	-2.83	**0.0199 (W)**
193_785.653	100520270.091 (128701810.336)	36706130.592 (61880351.239)	Down	-2.74	**0.0217 (W)**
331_111.091	3650915.773 (8465631.277)	944097.632 (787924.674)	Down	-3.87	**0.0220 (W)**
47_414.300	13052907.101 (20506448.245)	24781001.322 (29952933.001)	Up	1.9	**0.0220 (W)**
418_241.172	2502168.678 (0.000)	19626830.443 (51734485.009)	Up	7.84	**0.0234**
271_782.567	5227276.512 (9827526.950)	2031781.954 (5632134.017)	Down	-2.57	**0.0237 (W)**
200_822.565	7898671.939 (21082248.905)	935677.714 (490091.894)	Down	-8.44	**0.0250 (W)**
488_810.597	34936777.114 (101016586.666)	7351947.379 (45767968.857)	Down	-4.75	**0.0251 (W)**
578_111.091	2440431.307 (4870118.232)	849854.916 (0.000)	Down	-2.87	**0.0252**
242_647.512	9765315.744 (12782677.619)	5010083.202 (7430822.200)	Down	-1.95	**0.0259 (W)**
208_584.397	2690831.824 (0.000)	22783766.434 (62461480.394)	Up	8.47	**0.0273**
120_824.578	810467.676 (0.000)	2672877.527 (5818854.663)	Up	3.3	**0.0281**
227_818.590	954390.285 (0.000)	7737895.196 (21309022.603)	Up	8.11	**0.0289**
542_814.688	5444154.849 (21197141.716)	1119748.826 (2415743.552)	Down	-4.86	**0.0290 (W)**
218_315.133	1055162.878 (721112.604)	3647435.631 (12488793.551)	Up	3.46	**0.0301 (W)**
519_535.400	3183862.926 (6760763.169)	1488605.978 (3712675.162)	Down	-2.14	**0.0315 (W)**
134_991.672	88221290.449 (57079344.253)	67910281.244 (50636106.535)	Down	-1.3	**0.0315 (W)**
210_288.216	10868669.094 (11200608.839)	8632410.209 (15518565.014)	Down	-1.26	**0.0335 (W)**
324_762.593	4430952.181 (11657777.044)	841220.118 (0.000)	Down	-5.27	**0.0343**
489_752.559	14748580.588 (45374055.551)	4879746.592 (18175774.253)	Down	-3.02	**0.0347 (W)**
419_280.262	982998.940 (0.000)	9744688.955 (28641090.861)	Up	9.91	**0.0354**
297_522.355	41133125.047 (58065455.355)	25184487.195 (47447490.538)	Down	-1.63	**0.0356 (W)**
81_450.321	7434028.523 (12912154.330)	14424574.535 (20162499.020)	Up	1.94	**0.0362 (W)**
76_286.143	36705304.869 (82568478.475)	56712237.367 (84532836.151)	Up	1.55	**0.0366 (W)**
311_112.087	83308127.406 (542166891.729)	4730185.283 (4658433.148)	Down	-17.61	**0.0376 (W)**
428_585.270	5624430.329 (7806266.065)	3697727.996 (5986093.679)	Down	-1.52	**0.0413 (W)**
330_830.568	25955056.744 (30440348.953)	14041373.075 (22612966.401)	Down	-1.85	**0.0418 (W)**
341_564.463	4843236.060 (6068098.482)	3638257.117 (7316698.979)	Down	-1.33	**0.0418 (W)**
321_111.091	11753738.233 (30204105.789)	3592868.706 (9490873.883)	Down	-3.27	**0.0418 (W)**
167_746.606	24783878.889 (59482371.835)	49366430.046 (83890262.434)	Up	1.99	**0.0437 (W)**
361_878.700	11315549.650 (35674930.405)	928819.843 (0.000)	Down	-12.18	**0.0449**
316_111.0918	7332276.730 (9553602.684)	3907212.428 (7495659.827)	Down	-1.88	**0.0453 (W)**

369_1254.478	1606055.049 (1582628.584)	1149509.249 (0.000)	Down	-1.4	**0.0468**
574_1307.950	1858891.987 (3100450.968)	965073.827 (0.000)	Down	-1.93	**0.0469**
511_1254.521	1495868.714 (1513863.805)	1062796.574 (0.000)	Down	-1.41	**0.0486**
546_355.0644	204795566.557 (222828577.480)	136119673.613 (84733825.722)	Down	-1.5	**0.0487 (W)**
196_790.559	819125.628 (0.000)	4036736.332 (11275236.268)	Up	4.93	**0.0491**
482_1303.683	1618765.851 (2223985.153)	949959.228 (651675.913)	Down	-1.7	0.0505 (W)
548_804.552	4161006.234 (11149158.690)	1097403.429 (2012556.169)	Down	-3.79	0.0505 (W)
269_774.601	6635652.593 (15370759.740)	27686527.363 (59773138.681)	Up	4.17	0.0515 (W)
5_544.339	890043650.708 (222234164.293)	983924023.902 (253223025.531)	Up	1.11	0.0516
524_645.465	1951749.314 (3970024.611)	1024295.961 (1477744.287)	Down	-1.91	0.0523 (W)
565_815.697	1849818.782 (4521583.228)	870933.885 (488606.673)	Down	-2.12	0.0523 (W)
57_494.324	30457411.092 (22084458.366)	39732362.574 (23796733.791)	Up	1.3	0.0527 (W)
110_731.606	205404001.882 (210635700.484)	142189486.413 (214408265.985)	Down	-1.44	0.0531 (W)
436_112.087	6380122.560 (13437198.405)	1999542.234 (3315282.958)	Down	-3.19	0.0547 (W)
325_872.770	13539219.227 (74862948.607)	2534481.974 (8589273.504)	Down	-5.34	0.0557 (W)
157_813.684	5711518.775 (33538263.707)	16484955.679 (58298908.935)	Up	2.89	0.0562 (W)
388_690.470	931624.283 (0.000)	2101763.640 (4264401.890)	Up	2.26	0.0581
63_482.324	33376783.768 (14198243.991)	28640804.656 (12574716.270)	Down	-1.17	0.0584 (W)
241_818.590	1089003.064 (0.000)	4464561.380 (12357592.873)	Up	4.1	0.0592
584_111.091	1608251.059 (2900944.703)	815985.694 (0.000)	Down	-1.97	0.0593
186_815.698	2970416.490 (6197052.433)	1126528.848 (1267190.771)	Down	-2.64	0.0600 (W)
52_376.259	55533929.608 (16041947.547)	50817780.117 (15684309.944)	Down	-1.09	0.0603 (W)
37_548.371	92118605.054 (74680786.529)	64212618.188 (22415566.089)	Down	-1.43	0.0622 (W)
202_524.371	225023248.216 (240119764.883)	322130864.372 (249228056.573)	Up	1.43	0.0631 (W)
239_177.102	12512504.431 (36716662.890)	30493313.479 (72446722.778)	Up	2.44	0.0637 (W)
383_874.783	2200855.286 (4993161.751)	861567.351 (0.000)	Down	-2.55	0.0638
467_557.413	3711767.417 (8358495.073)	1708039.796 (4333748.859)	Down	-2.17	0.0688 (W)
314_111.091	5250159.291 (8965096.627)	2363894.151 (4315087.293)	Down	-2.22	0.0691 (W)
213_595.492	25727015.808 (54353255.238)	38608954.981 (67975291.375)	Up	1.5	0.0693 (W)
350_535.399	1548643.820 (1948512.024)	3389131.009 (6251867.939)	Up	2.19	0.0695 (W)
335_797.624	2354628.618 (3615054.386)	3404808.001 (13735370.976)	Up	1.45	0.0713 (W)
411_786.600	10551882.795 (29802327.611)	16576916.791 (32216847.996)	Up	1.57	0.0723 (W)
278_772.622	38908284.738 (67939883.110)	14837248.276 (35596557.254)	Down	-2.62	0.0756 (W)
400_355.063	3470260.643 (7209440.795)	1519277.572 (2495536.784)	Down	-2.28	0.0766 (W)
50_633.255	10906199.118 (15792157.969)	15266143.568 (38246105.273)	Up	1.4	0.0778 (W)
340_558.472	6410047.200 (7961341.401)	13956245.006 (18375892.121)	Up	2.18	0.0808 (W)
484_1041.679	3145306.838 (3846456.468)	2202239.422 (3583012.344)	Down	-1.43	0.0809 (W)
255_834.597	9099005.404 (22769567.985)	3528607.429 (7244314.588)	Down	-2.58	0.0814 (W)
491_874.784	10983877.489 (49796934.860)	965261.378 (743240.848)	Down	-11.38	0.0814 (W)
500_112.087	3554306.395 (6619471.437)	1473256.653 (2694423.659)	Down	-2.41	0.0820 (W)
462_1231.336	1368339.891 (1159604.608)	1079303.854 (0.000)	Down	-1.27	0.0842
323_1167.807	1343269.738 (1146600.418)	1057871.727 (0.000)	Down	-1.27	0.0846
512_1189.808	1471976.658 (1263099.505)	1157636.176 (0.000)	Down	-1.27	0.0847
536_834.608	3262355.460 (0.000)	4185070.064 (3711809.754)	Up	1.28	0.085
315_1207.783	1231074.843 (1105138.556)	956624.677 (0.000)	Down	-1.29	0.0853
23_508.376	166030413.612 (80716082.014)	151441106.859 (86382886.329)	Down	-1.1	0.0854 (W)
461_1209.028	1451566.289 (1289582.085)	1132101.339 (0.000)	Down	-1.28	0.0861
346_284.115	2069644.737 (1084044.317)	4580622.761 (9085887.535)	Up	2.21	0.0876 (W)
424_355.262	955712.031 (500585.525)	3535802.768 (8942773.052)	Up	3.7	0.0876 (W)
499_787.668	2473932.072 (5756204.029)	974505.637 (510429.292)	Down	-2.54	0.0876 (W)
506_1208.117	1236740.519 (1138691.233)	956225.217 (0.000)	Down	-1.29	0.0878
450_797.054	11331264.132 (0.000)	14674854.248 (13620395.048)	Up	1.3	0.0889
176_782.567	1752428.133 (4913385.514)	3683173.638 (11855166.803)	Up	2.1	0.0889 (W)
320_1148.468	1155409.562 (1117676.482)	882509.936 (0.000)	Down	-1.31	0.0906
207_800.577	3442966.264 (2355821.319)	38118537.994 (120880365.477)	Up	11.07	0.0907 (W)
355_1146.433	1177333.729 (0.000)	1541853.797 (1494307.667)	Up	1.31	0.0908
155_212.019	977932.754 (0.000)	8005744.406 (28845602.160)	Up	8.19	0.0912
520_579.426	3567330.592 (8111079.760)	1877989.217 (4854859.243)	Down	-1.9	0.0916 (W)
521_623.452	2630539.828 (5158844.394)	1598370.777 (3327435.088)	Down	-1.65	0.0916 (W)
569_111.091	2141278.156 (1997056.349)	1655755.882 (0.000)	Down	-1.29	0.0919
274_770.606	26720281.235 (41894853.749)	12368162.649 (26273060.142)	Down	-2.16	0.0923 (W)
568_1148.224	1090791.444 (1060722.081)	833437.978 (0.000)	Down	-1.31	0.0926
446_721.337	15813683.354 (0.000)	20682029.646 (20108860.609)	Up	1.31	0.0932
270_834.597	5834270.189 (23591537.606)	1144150.736 (1583998.463)	Down	-5.1	0.0940 (W)
463_1209.667	1253945.519 (1225346.143)	958185.748 (0.000)	Down	-1.31	0.0942
291_796.620	4061618.614 (19733354.822)	7283794.139 (31129391.984)	Up	1.79	0.0943 (W)
457_658.699	2915961.690 (0.000)	3993617.000 (4467785.407)	Up	1.37	0.0944
441_757.201	8021543.780 (0.000)	10903961.917 (12057969.506)	Up	1.36	0.0973
571_872.770	1558637.025 (3339358.635)	761515.763 (0.000)	Down	-2.05	0.0978
448_721.240	13092284.914 (0.000)	18870021.821 (24349210.407)	Up	1.44	0.0997
532_785.653	39328270.182 (116598960.041)	6385255.235 (35976430.914)	Down	-6.16	0.1003 (W)
453_756.497	3527205.356 (0.000)	5092198.881 (6624988.520)	Up	1.44	0.1012
554_455.037	338884827.400 (0.000)	456992803.896 (501459330.104)	Up	1.35	0.1022
445_721.289	11746652.884 (0.000)	16107116.759 (18521533.317)	Up	1.37	0.1024

14_188.070	1594754348.754 (333836151.005)	1450225051.024 (404075296.063)	Down	-1.1	0.1030 (W)
559_323.055	32456271.640 (0.000)	44176699.348 (50060624.513)	Up	1.36	0.1042
510_854.569	1258838.890 (1450657.416)	956680.830 (1086578.443)	Down	-1.32	0.1043 (W)
364_898.785	16080877.721 (65625414.333)	763269.231 (0.000)	Down	-21.07	0.1052
82_556.457	32194218.334 (19562853.748)	26536477.566 (20365978.395)	Down	-1.21	0.1059 (W)
245_830.554	864703.231 (0.000)	2023800.177 (4987797.840)	Up	2.34	0.1067
421_563.551	1140258.428 (0.000)	2459057.009 (5677859.139)	Up	2.16	0.1069
456_688.502	7686946.148 (0.000)	10388638.293 (11645953.535)	Up	1.35	0.1073
225_810.599	1493191.833 (2877319.457)	9812546.568 (54231192.331)	Up	6.57	0.1084 (W)
67_508.339	16183009.987 (10935417.615)	20085575.122 (12520856.547)	Up	1.24	0.1089 (W)
583_112.087	1475786.564 (2081184.661)	996027.974 (0.000)	Down	-1.48	0.1095
217_810.598	1443995.322 (2366913.444)	15159659.391 (55544982.838)	Up	10.5	0.1099 (W)
247_810.599	45453047.824 (57470032.923)	27595795.280 (44054408.155)	Down	-1.65	0.1131 (W)
216_610.504	56159010.595 (62599726.721)	45084413.771 (54464440.389)	Down	-1.25	0.1134 (W)
451_721.192	10109693.334 (0.000)	14686120.081 (20110781.184)	Up	1.45	0.114
556_235.180	60114569.460 (0.000)	84075085.094 (105318732.901)	Up	1.4	0.1141
443_688.549	9603639.428 (0.000)	13444770.665 (16894859.539)	Up	1.4	0.1143
447_797.161	6008604.582 (0.000)	8473159.085 (10867946.040)	Up	1.41	0.1152
308_718.574	9238804.421 (34290221.514)	1470429.147 (0.000)	Down	-6.28	0.1156
444_757.252	9780039.398 (0.000)	13738742.736 (17494628.648)	Up	1.4	0.116
385_1165.781	1451179.395 (2079286.286)	981722.164 (0.000)	Down	-1.48	0.1168
237_124.087	23514619.492 (15122797.436)	26712320.592 (17948429.212)	Up	1.14	0.1172 (W)
439_1231.346	6719081.178 (0.000)	11115430.622 (19590701.754)	Up	1.65	0.119
454_797.107	5290765.244 (0.000)	7924271.111 (11757220.363)	Up	1.5	0.1197
498_513.387	3039324.542 (6106857.188)	1742644.693 (3678512.269)	Down	-1.74	0.1200 (W)
229_358.295	1431295.290 (1981022.450)	5108191.196 (15544051.569)	Up	3.57	0.1228 (W)
442_757.302	11151874.998 (0.000)	16992809.219 (26311864.170)	Up	1.52	0.1229
493_256.299	1205036.724 (0.000)	5802287.697 (20751164.220)	Up	4.82	0.1237
106_682.363	4281475.193 (8695296.731)	4150711.862 (5548270.173)	Down	-1.03	0.1260 (W)
7_494.324	463381194.102 (163826721.262)	515792308.533 (175699408.675)	Up	1.11	0.1261
187_279.231	833491.681 (0.000)	3978717.528 (14311260.075)	Up	4.77	0.1266
194_112.087	4705255.630 (13577535.206)	3675768.422 (5182483.203)	Down	-1.28	0.1277 (W)
108_787.667	4048900.171 (11897093.417)	5332761.453 (11264744.103)	Up	1.32	0.1277 (W)
455_794.373	3350402.844 (0.000)	5011771.036 (7595645.132)	Up	1.5	0.1284
131_808.582	39685048.507 (110145537.310)	7994750.580 (22927292.128)	Down	-4.96	0.1322 (W)
585_1231.200	1713854.652 (2397160.414)	1197623.616 (0.000)	Down	-1.43	0.1342
558_909.066	21439341.920 (0.000)	32181097.895 (50067937.325)	Up	1.5	0.1357
513_376.175	2922719.052 (0.000)	5877089.555 (13776793.907)	Up	2.01	0.1359
221_336.289	1091468.361 (0.000)	3880356.785 (13010986.487)	Up	3.56	0.136
555_265.203	31792896.120 (0.000)	74670992.805 (200276918.443)	Up	2.35	0.1365
515_387.179	2692634.302 (0.000)	6721374.144 (18830314.276)	Up	2.5	0.1367
514_359.148	2661409.278 (0.000)	5365538.937 (12660910.402)	Up	2.02	0.1374
191_830.554	1896541.252 (0.000)	3574155.081 (7855543.648)	Up	1.88	0.1374
557_529.399	5430562.932 (0.000)	13440693.169 (37611511.217)	Up	2.48	0.1385
124_815.697	12326473.031 (60857596.426)	2013416.142 (4736326.510)	Down	-6.12	0.1388 (W)
223_536.406	1181249.171 (2043830.580)	2193136.633 (4589131.344)	Up	1.86	0.1408 (W)
531_796.621	2368290.936 (4830471.834)	1295848.879 (2065062.333)	Down	-1.83	0.1408 (W)
458_1249.357	994933.058 (0.000)	2142286.575 (5458373.616)	Up	2.15	0.1436
378_111.091	12295394.067 (36572263.466)	3085088.729 (7786348.938)	Down	-3.99	0.1456 (W)
572_814.687	5835721.962 (20000884.822)	1682854.678 (0.000)	Down	-3.47	0.1484
367_874.785	8059128.458 (34605317.229)	875368.502 (0.000)	Down	-9.21	0.1485
509_872.770	29567064.206 (139099765.914)	761157.390 (0.000)	Down	-38.84	0.1495
198_796.621	121098130.748 (201176919.514)	51275784.674 (134116157.360)	Down	-2.36	0.1496 (W)
55_798.564	5034080.195 (16432646.809)	117816409.606 (232457001.005)	Up	23.4	0.1511 (W)
115_583.255	3394941.211 (5071581.506)	6460046.454 (14589963.232)	Up	1.9	0.1616 (W)
375_874.784	6065196.122 (29755392.449)	4741364.560 (21569899.167)	Down	-1.28	0.1617 (W)
145_731.606	56868351.446 (136122116.340)	29478244.485 (82622859.402)	Down	-1.93	0.1618 (W)
516_404.206	1027547.475 (0.000)	3067738.717 (10284743.704)	Up	2.99	0.167
394_1329.638	1559125.954 (1796068.839)	1154763.550 (733387.546)	Down	-1.35	0.1682 (W)
553_667.478	1739146.981 (3397152.269)	967841.371 (520924.226)	Down	-1.8	0.1682 (W)
537_111.091	5990864.036 (13825302.088)	2739355.454 (6306585.187)	Down	-2.19	0.1692 (W)
212_612.519	43792411.974 (81814603.250)	69605317.066 (109405900.050)	Up	1.59	0.1699 (W)
486_752.558	17379855.456 (42525772.961)	13049687.620 (45535035.926)	Down	-1.33	0.1703 (W)
401_815.697	1460715.774 (2372177.222)	1365391.509 (3063517.855)	Down	-1.07	0.1707 (W)
16_1047.735	109021805.297 (120798380.151)	64046920.661 (64234377.782)	Down	-1.7	0.1712 (W)
452_756.544	2517772.950 (0.000)	4322622.621 (9211144.125)	Up	1.72	0.1722
182_813.685	2576571.196 (12556264.369)	37517702.859 (200016902.254)	Up	14.56	0.1739 (W)
371_1278.615	2512844.341 (7438397.203)	1262991.969 (711755.476)	Down	-1.99	0.1739 (W)
224_810.599	1299249.467 (2871841.145)	2361629.279 (7096584.490)	Up	1.82	0.1797 (W)
327_1145.614	1280308.599 (1760852.711)	950222.526 (926225.643)	Down	-1.35	0.1797 (W)
577_112.087	2045338.913 (3802345.708)	1419118.858 (1788542.012)	Down	-1.44	0.1797 (W)
132_814.687	7798498.720 (20422118.001)	4559803.132 (11474115.764)	Down	-1.71	0.1815 (W)
449_757.352	2302498.952 (0.000)	4390239.252 (10948775.519)	Up	1.91	0.1838
497_601.439	3108379.211 (6431722.840)	1750972.518 (3853188.378)	Down	-1.78	0.1854 (W)

204_166.086	316007366.768 (598672893.416)	464468668.712 (693453277.370)	Up	1.47	0.1894 (W)
508_814.687	6187761.607 (26226298.681)	2973387.054 (12010838.755)	Down	-2.08	0.1899 (W)
101_539.430	34505998.513 (31392793.341)	27234208.677 (22309710.095)	Down	-1.27	0.1922 (W)
209_344.279	20165490.321 (15652484.737)	17543383.700 (18797024.549)	Down	-1.15	0.1958 (W)
539_796.621	46790230.022 (109610703.131)	29646700.539 (97507258.671)	Down	-1.58	0.1969 (W)
292_814.686	3598309.696 (8962341.731)	5472994.821 (12255558.890)	Up	1.52	0.2043 (W)
201_136.087	3969510.833 (2341361.182)	3092192.938 (2534533.605)	Down	-1.28	0.2046 (W)
97_469.388	35600560.126 (18651748.392)	32844993.853 (27554407.448)	Down	-1.08	0.2108 (W)
342_617.474	841532.950 (0.000)	8531364.745 (43021311.247)	Up	10.14	0.2122
343_563.428	880926.438 (0.000)	3414657.385 (14228569.635)	Up	3.88	0.2139
235_812.615	1662380.516 (0.000)	32918192.234 (176984933.836)	Up	19.8	0.2177
234_612.520	3284183.027 (4711986.616)	7556685.681 (30468351.870)	Up	2.3	0.2187 (W)
406_520.339	1555874.291 (2138190.113)	2060470.393 (2658195.336)	Up	1.32	0.2288 (W)
250_355.063	541975237.538 (363660223.046)	491728562.646 (359696223.381)	Down	-1.1	0.2338 (W)
395_537.414	1149285.453 (938788.291)	1418398.000 (1397326.149)	Up	1.23	0.2456 (W)
68_245.186	4607464.967 (5906570.552)	21587084.510 (31268050.792)	Up	4.69	0.2500 (W)
459_810.598	809214.187 (0.000)	13029169.583 (75863565.700)	Up	16.1	0.2602
80_810.598	239985420.160 (253185241.761)	281983820.181 (284410687.942)	Up	1.18	0.2637 (W)
338_814.687	1392002.632 (2156324.048)	1236716.363 (2684288.106)	Down	-1.13	0.2651 (W)
283_814.687	14012237.567 (38593201.634)	5637652.536 (9518398.186)	Down	-2.49	0.2692 (W)
107_519.404	19385016.007 (33916596.627)	15108536.480 (17844976.002)	Down	-1.28	0.2777 (W)
159_808.576	3526529.788 (3914191.951)	12145487.657 (33389153.467)	Up	3.44	0.2804 (W)
135_123.079	5215294.394 (6729334.412)	4078429.358 (5627544.279)	Down	-1.28	0.2828 (W)
244_330.263	1168424.395 (0.000)	5472297.915 (28100780.898)	Up	4.68	0.2841
109_466.316	9583255.810 (54302820.234)	4061343.655 (8086160.892)	Down	-2.36	0.2891 (W)
430_212.118	26681781.880 (151854084.992)	2172797.897 (4396486.264)	Down	-12.28	0.2911 (W)
586_718.575	8405122.189 (42578843.843)	2048197.192 (0.000)	Down	-4.1	0.2963
582_744.552	4493689.919 (18614922.864)	1724019.861 (0.000)	Down	-2.61	0.2979
280_1166.462	1690106.191 (1872959.268)	1330115.851 (696691.806)	Down	-1.27	0.3004 (W)
357_1126.926	1591879.539 (1724270.899)	1275077.456 (667863.641)	Down	-1.25	0.3004 (W)
440_657.202	2444190.591 (1280224.994)	7540736.181 (21646052.758)	Up	3.09	0.3004 (W)
465_1329.728	1424351.463 (1434229.888)	1155633.994 (605301.210)	Down	-1.23	0.3004 (W)
561_1186.593	1609780.099 (2259413.160)	1134606.556 (594287.400)	Down	-1.42	0.3004 (W)
579_874.784	13602714.887 (75515220.909)	2465511.452 (0.000)	Down	-5.52	0.3021
184_370.294	27588839.881 (27179702.181)	27210674.198 (31640983.468)	Down	-1.01	0.3043 (W)
352_614.534	5122635.704 (13115262.019)	13079070.628 (34688281.693)	Up	2.55	0.3048 (W)
188_731.606	9743131.054 (21091912.802)	5148549.832 (14223566.588)	Down	-1.89	0.3092 (W)
580_787.668	7475425.774 (38594316.808)	1867000.126 (0.000)	Down	-4	0.3092
166_1385.157	1248533.488 (906773.850)	1555273.690 (1839138.792)	Up	1.25	0.3100 (W)
359_480.344	10270796.229 (15823530.437)	7611523.543 (5194778.162)	Down	-1.35	0.3100 (W)
374_1148.528	1226259.842 (1310998.281)	983080.162 (536127.181)	Down	-1.25	0.3100 (W)
404_630.530	849253.024 (453035.734)	2942790.196 (13306058.367)	Up	3.47	0.3100 (W)
470_1256.674	1154033.422 (713728.585)	1416450.030 (1490421.944)	Up	1.23	0.3100 (W)
483_1256.771	1348621.916 (1865447.859)	1019879.431 (587318.536)	Down	-1.32	0.3100 (W)
538_643.379	2657630.453 (8319158.604)	1376946.464 (746015.413)	Down	-1.93	0.3100 (W)
566_731.606	1157149.109 (1341903.346)	920407.668 (483369.000)	Down	-1.26	0.3100 (W)
416_382.978	861274.405 (0.000)	9941565.281 (63139613.505)	Up	11.54	0.3142
540_774.601	13461421.915 (34651144.407)	9318567.921 (28646488.701)	Down	-1.44	0.3165 (W)
487_112.086	7101097.139 (14455485.721)	2928858.919 (7822434.170)	Down	-2.42	0.3195 (W)
173_813.681	2038303.342 (3477172.836)	10293056.131 (59665686.517)	Up	5.05	0.3198 (W)
306_780.553	2908338.207 (4584076.927)	2175654.080 (1480401.770)	Down	-1.34	0.3198 (W)
362_1208.896	1488346.164 (1399297.684)	1255844.308 (777918.258)	Down	-1.19	0.3198 (W)
381_1108.126	981765.849 (580366.109)	1185577.047 (1201014.825)	Up	1.21	0.3198 (W)
563_872.770	1588829.741 (3460604.569)	1243598.082 (2718142.596)	Down	-1.28	0.3198 (W)
137_321.242	1992612.945 (6155326.136)	2184684.906 (9129565.609)	Up	1.1	0.3297 (W)
386_1280.911	3310075.374 (15343488.302)	1508721.239 (1499491.967)	Down	-2.19	0.3297 (W)
130_814.687	2501609.807 (5286833.544)	4753206.423 (9454725.460)	Up	1.9	0.3305 (W)
215_342.263	10979020.553 (13771770.769)	10355319.949 (16439231.333)	Down	-1.06	0.3309 (W)
431_1102.058	2585050.672 (7151834.951)	2628609.466 (5309279.058)	Up	1.02	0.3345 (W)
413_280.263	2920792.233 (8105677.386)	3684652.626 (17139167.207)	Up	1.26	0.3380 (W)
70_468.308	17909171.528 (13802203.431)	19954767.445 (14134523.163)	Up	1.11	0.3394 (W)
185_787.668	11655287.238 (55509541.299)	50668314.848 (172086848.881)	Up	4.35	0.3484 (W)
197_111.091	10658310.596 (19760486.040)	4002533.573 (6258774.442)	Down	-2.66	0.3497 (W)
478_772.621	10977987.665 (27847972.523)	2604854.400 (5014167.556)	Down	-4.21	0.3501 (W)
162_815.697	11033068.364 (27689583.318)	4126613.005 (12214852.665)	Down	-2.67	0.3516 (W)
170_786.600	97843730.305 (164177983.968)	44899937.613 (65710887.235)	Down	-2.18	0.3519 (W)
125_731.606	1595196.300 (2450488.227)	2966139.408 (7325071.428)	Up	1.86	0.3668 (W)
205_120.080	214728197.146 (470291737.552)	281377919.360 (514412972.024)	Up	1.31	0.3706 (W)
59_510.391	36502511.228 (19358129.858)	36332335.925 (25151403.352)	Down	-1	0.3720 (W)
38_290.269	86182526.894 (9744822.095)	88540687.225 (10547484.345)	Up	1.03	0.3832 (W)
199_787.604	1409102.339 (2030494.186)	2163485.615 (4808816.441)	Up	1.54	0.3859 (W)
24_205.097	1051985661.842 (194717492.128)	989439854.527 (273961538.428)	Down	-1.06	0.3869 (W)
545_111.091	2043527.322 (5239344.208)	1173396.882 (2228228.626)	Down	-1.74	0.3884 (W)
228_519.404	8500518.425 (8785207.499)	9368891.110 (13401241.337)	Up	1.1	0.3915 (W)

522_491.374	2254687.568 (4901298.628)	1591588.092 (2879106.086)	Down	-1.42	0.3977 (W)
412_754.538	2119294.282 (5085989.819)	1783078.639 (5432565.519)	Down	-1.19	0.4071 (W)
434_979.945	1424482.643 (3652047.832)	1556412.338 (2902178.677)	Up	1.09	0.4071 (W)
505_1329.734	1279134.390 (1225088.804)	1101239.396 (870946.297)	Down	-1.16	0.4071 (W)
12_468.308	241676556.606 (108890807.638)	255591902.093 (114390615.289)	Up	1.06	0.4179 (W)
92_426.320	2726892.266 (5225221.552)	11241214.161 (28031869.768)	Up	4.12	0.4184 (W)
344_577.482	9765701.613 (24923525.351)	9579889.760 (23111462.008)	Down	-1.02	0.4211 (W)
74_1087.672	7607443.873 (6561962.362)	8937403.921 (7279503.865)	Up	1.17	0.4263 (W)
65_265.118	76595834.590 (65148329.888)	87974231.581 (68502964.032)	Up	1.15	0.4273 (W)
195_112.087	5809854.985 (20244836.655)	1677537.125 (2518659.484)	Down	-3.46	0.4311 (W)
233_562.421	1609921.193 (3495684.397)	1415424.321 (1720257.692)	Down	-1.14	0.4361 (W)
534_815.697	7853320.227 (20385289.819)	2501986.693 (7290445.879)	Down	-3.14	0.4416 (W)
46_521.420	118914475.306 (73115629.534)	108770233.993 (71447737.421)	Down	-1.09	0.4503 (W)
353_1166.578	1155530.824 (1269951.624)	1379497.685 (1652184.943)	Up	1.19	0.4549 (W)
243_838.631	37931302.485 (61759345.373)	52115111.787 (79874818.418)	Up	1.37	0.4696 (W)
433_979.721	1710742.540 (3836475.226)	2216779.155 (4511978.411)	Up	1.3	0.4727 (W)
286_814.687	39182372.342 (104693160.920)	24400893.199 (84833432.526)	Down	-1.61	0.4766 (W)
425_585.269	17879157.152 (38880520.309)	15810141.590 (32895892.119)	Down	-1.13	0.4957 (W)
122_808.581	12664889.901 (38886642.135)	7065236.780 (21305102.953)	Down	-1.79	0.4995 (W)
126_815.698	3085322.190 (8549723.594)	1830804.486 (3928194.402)	Down	-1.69	0.5037 (W)
26_120.080	845612027.997 (354259742.118)	808994792.156 (476096165.750)	Down	-1.05	0.5093 (W)
203_132.102	732740099.092 (791477086.016)	694521411.944 (910399878.411)	Down	-1.06	0.5165 (W)
501_226.182	2550254.192 (3956782.379)	2001676.394 (3112294.285)	Down	-1.27	0.5253 (W)
380_872.770	8953318.037 (52588462.340)	3852265.116 (17487953.663)	Down	-2.32	0.5293 (W)
289_814.687	7867669.696 (42058867.014)	3420684.485 (11016044.048)	Down	-2.3	0.5486 (W)
261_1127.219	1047579.425 (548704.085)	1312982.397 (1810803.087)	Up	1.25	0.5519 (W)
266_1145.300	1253163.986 (656385.743)	1373261.824 (1053721.972)	Up	1.1	0.5519 (W)
312_1127.035	1164033.621 (1183320.345)	1001037.440 (524326.194)	Down	-1.16	0.5519 (W)
390_1356.798	1421788.982 (744708.615)	1622677.162 (1589014.425)	Up	1.14	0.5519 (W)
477_1090.168	1138470.335 (1011916.911)	1008997.201 (528495.380)	Down	-1.13	0.5519 (W)
502_1306.108	1226255.644 (1671461.967)	988557.279 (517789.300)	Down	-1.24	0.5519 (W)
503_1186.913	1183741.609 (1221450.678)	1012034.280 (530086.150)	Down	-1.17	0.5519 (W)
517_444.281	15467131.600 (47273216.972)	7254042.759 (3799542.834)	Down	-2.13	0.5519 (W)
326_111.091	4689889.745 (10754517.342)	3010629.650 (5559735.454)	Down	-1.56	0.5538 (W)
153_399.250	5539361.883 (15966369.364)	7831836.314 (22057881.249)	Up	1.41	0.5573 (W)
103_585.270	371896831.767 (323154918.271)	310657894.138 (240221575.520)	Down	-1.2	0.5599 (W)
246_813.684	1040108.691 (1254055.936)	1236959.679 (2202717.761)	Up	1.19	0.5676 (W)
248_813.684	1780082.695 (1440388.161)	34125512.251 (229248913.108)	Up	19.17	0.5676 (W)
263_1186.517	1155541.514 (1065659.744)	1073665.408 (889199.963)	Down	-1.08	0.5676 (W)
267_1255.704	984009.976 (773577.150)	1057376.694 (929971.585)	Up	1.07	0.5676 (W)
275_1278.678	1326663.317 (947268.509)	1226834.578 (645891.887)	Down	-1.08	0.5676 (W)
345_1254.659	1261862.021 (890422.841)	1383441.640 (1269056.309)	Up	1.1	0.5676 (W)
351_1332.265	1080883.400 (1008960.106)	1007468.408 (867719.067)	Down	-1.07	0.5676 (W)
393_1356.874	1252871.506 (1006307.671)	1148294.955 (679390.077)	Down	-1.09	0.5676 (W)
397_1256.734	1109216.153 (842576.816)	1033802.417 (663027.806)	Down	-1.07	0.5676 (W)
399_1128.104	959727.687 (649556.583)	1151570.420 (1583142.545)	Up	1.2	0.5676 (W)
460_1387.659	1208644.124 (1164619.490)	1118647.838 (954577.440)	Down	-1.08	0.5676 (W)
464_1187.258	983836.899 (912467.648)	1057992.965 (1060072.703)	Up	1.08	0.5676 (W)
471_1414.423	1560240.467 (1156671.858)	1436969.711 (774499.074)	Down	-1.09	0.5676 (W)
476_1385.196	1026255.580 (660003.194)	1146108.570 (1103322.765)	Up	1.12	0.5676 (W)
480_1448.041	1066322.064 (1084554.130)	986662.934 (902241.755)	Down	-1.08	0.5676 (W)
507_1231.382	1456533.899 (2863247.476)	1078975.773 (704716.664)	Down	-1.35	0.5676 (W)
518_606.369	1869485.667 (7077705.508)	1453110.283 (4583451.266)	Down	-1.29	0.5676 (W)
525_520.339	2537499.144 (10055083.263)	1246459.401 (1505633.948)	Down	-2.04	0.5676 (W)
526_564.359	1993086.793 (6954276.119)	1031103.205 (672158.982)	Down	-1.93	0.5676 (W)
143_811.668	95431232.629 (153215122.546)	76364567.312 (111717041.473)	Down	-1.25	0.5750 (W)
347_521.420	2985964.953 (4935374.498)	8920122.344 (33784388.061)	Up	2.99	0.5825 (W)
494_814.687	22817654.088 (66784843.945)	20522758.146 (50930087.750)	Down	-1.11	0.5826 (W)
260_1110.151	1412272.394 (1833174.950)	1348082.855 (1897823.502)	Down	-1.05	0.5836 (W)
268_1108.211	1471926.146 (1576925.034)	1562484.801 (1654695.759)	Up	1.06	0.5836 (W)
329_1125.575	1260626.354 (905172.530)	1195092.786 (827910.259)	Down	-1.05	0.5836 (W)
348_1129.053	1161263.028 (910122.522)	1115077.218 (960874.731)	Down	-1.04	0.5836 (W)
372_1188.152	1113297.089 (935295.498)	1146712.554 (1546117.278)	Up	1.03	0.5836 (W)
391_1209.102	1219005.466 (966824.889)	1152641.637 (896284.530)	Down	-1.06	0.5836 (W)
405_824.574	2801536.397 (12230744.488)	1511782.011 (2564770.677)	Down	-1.85	0.5836 (W)
469_1331.925	1304862.575 (1083037.664)	1253197.979 (1154318.148)	Down	-1.04	0.5836 (W)
479_1089.328	1098746.963 (932068.901)	1048371.632 (947056.645)	Down	-1.05	0.5836 (W)
481_1146.356	1037147.563 (967981.959)	973014.430 (877066.300)	Down	-1.07	0.5836 (W)
504_1256.647	1007768.151 (719268.734)	982025.457 (844418.306)	Down	-1.03	0.5836 (W)
549_1487.022	1265515.382 (714158.248)	1351378.879 (924724.757)	Up	1.07	0.5836 (W)
550_593.441	873065.489 (670254.666)	1471177.126 (5179796.218)	Up	1.69	0.5836 (W)
551_571.429	1003027.368 (1142496.811)	1478573.348 (4870084.006)	Up	1.47	0.5836 (W)
552_505.390	1074636.447 (1162043.091)	1330353.127 (3358497.808)	Up	1.24	0.5836 (W)
576_1233.440	1624376.916 (1167837.554)	1563263.950 (1233754.011)	Down	-1.04	0.5836 (W)

336_780.553	8750336.387 (17462600.247)	7027657.110 (17999481.112)	Down	-1.25	0.6063 (W)
389_800.575	2287426.191 (3593758.568)	21101371.991 (79802453.744)	Up	9.22	0.6157 (W)
420_256.263	3439529.784 (5804083.601)	5049100.001 (14906769.453)	Up	1.47	0.6269 (W)
402_1254.378	1178066.045 (1198682.699)	1042104.556 (815021.681)	Down	-1.13	0.6286 (W)
20_166.086	1154065466.481 (426656833.402)	1096320732.101 (630944859.215)	Down	-1.05	0.6329 (W)
232_593.477	7982637.634 (15141963.534)	8345968.718 (18516328.013)	Up	1.05	0.6351 (W)
387_731.606	92442040.198 (219167141.786)	69122698.569 (140337204.303)	Down	-1.34	0.6370 (W)
272_1278.643	1567072.305 (1972171.054)	1380658.520 (1529499.789)	Down	-1.14	0.6416 (W)
282_1148.410	1157429.166 (941844.472)	1304018.621 (1399897.425)	Up	1.13	0.6416 (W)
368_1166.548	1554903.129 (1531028.033)	1400510.674 (1131485.884)	Down	-1.11	0.6416 (W)
472_1329.711	1467831.459 (1477751.546)	1309804.095 (1022453.890)	Down	-1.12	0.6416 (W)
189_815.697	3569728.864 (7045170.847)	7558826.027 (32748244.967)	Up	2.12	0.6428 (W)
535_815.697	7437021.878 (25802944.439)	3824658.562 (7967845.779)	Down	-1.94	0.6470 (W)
116_1290.631	5232522.351 (10287768.742)	4165670.755 (6811551.807)	Down	-1.26	0.6540 (W)
34_469.388	176999984.728 (73874318.328)	180075123.453 (107119272.414)	Up	1.02	0.6566 (W)
382_593.477	10886815.009 (24698144.598)	12360182.286 (22518420.430)	Up	1.14	0.6644 (W)
485_112.087	11200622.274 (29880376.787)	3504138.100 (7987391.191)	Down	-3.2	0.6679 (W)
333_1166.639	1259756.157 (1842042.702)	1170420.673 (1506792.531)	Down	-1.08	0.6680 (W)
475_1092.115	1334529.584 (1201134.022)	1254548.440 (1071522.556)	Down	-1.06	0.6680 (W)
377_810.597	7381835.090 (27456819.838)	12334514.435 (41461535.450)	Up	1.67	0.6682 (W)
222_491.373	3112750.550 (4051948.638)	4880450.299 (7882650.231)	Up	1.57	0.6770 (W)
547_804.553	1206125.119 (1568263.251)	1552202.854 (3995070.596)	Up	1.29	0.6813 (W)
495_814.687	1962067.812 (4998516.307)	1465489.130 (3218622.849)	Down	-1.34	0.6911 (W)
527_527.401	1101442.628 (957813.187)	1712674.786 (4888629.093)	Up	1.55	0.6947 (W)
299_299.138	101526551.013 (58752277.594)	96441384.653 (77007570.147)	Down	-1.05	0.7015 (W)
438_838.632	5419045.658 (16854833.211)	6709095.333 (22173118.425)	Up	1.24	0.7025 (W)
1_496.339	9139207662.800 (1986811523.043)	9155973229.120 (1576907065.758)	Up	1	0.7071 (W)
62_471.737	5912090.927 (7429590.513)	18775162.912 (27182450.915)	Up	3.18	0.7094 (W)
496_814.687	2130240.154 (4905921.759)	1437656.414 (2244664.050)	Down	-1.48	0.7129 (W)
171_815.698	21105858.689 (111857403.180)	7532143.704 (24152903.648)	Down	-2.8	0.7142 (W)
354_539.430	1854368.318 (2139250.383)	2342995.662 (3308341.612)	Up	1.26	0.7329 (W)
358_112.086	7330638.356 (17546455.971)	3298258.094 (7556178.365)	Down	-2.22	0.7536 (W)
36_393.285	79823252.707 (23621345.707)	80648961.605 (24701164.981)	Up	1.01	0.7538 (W)
129_814.688	2297406.836 (7824873.884)	1654212.395 (2800208.977)	Down	-1.39	0.7547 (W)
104_760.585	82685481.170 (68230191.115)	80712454.142 (76468109.258)	Down	-1.02	0.7567 (W)
339_1169.533	10983140.514 (9922352.040)	13147610.583 (13812045.869)	Up	1.2	0.7680 (W)
211_239.091	17152036.318 (19198682.337)	23628500.764 (36577744.240)	Up	1.38	0.7685 (W)
15_482.360	181431495.320 (90793821.973)	195961013.614 (84919247.272)	Up	1.08	0.8227 (W)
169_495.404	24188661.177 (18805356.735)	28817506.246 (31038662.985)	Up	1.19	0.8278 (W)
172_787.667	5456586.951 (13858250.043)	7171274.566 (24695396.402)	Up	1.31	0.8287 (W)
19_510.355	337533239.661 (215370600.905)	357682297.083 (228724468.585)	Up	1.06	0.8388 (W)
136_181.0725	62644459.812 (107960005.844)	60224157.040 (83565473.653)	Down	-1.04	0.8466 (W)
206_282.278	14558679.980 (27375970.401)	119800697.343 (326121380.172)	Up	8.23	0.8710 (W)
181_786.601	1610236.796 (2569219.100)	1778050.385 (3306987.337)	Up	1.1	0.8735 (W)
179_787.668	92256800.722 (238422165.325)	60713745.597 (145247920.497)	Down	-1.52	0.8735 (W)
161_110.0713	2897452.896 (3761247.421)	2748698.869 (3039453.650)	Down	-1.05	0.8829 (W)
9_132.102	1207780357.622 (824081240.334)	1312388635.369 (871930514.016)	Up	1.09	0.8864 (W)
158_815.697	3513509.312 (13604220.284)	5413346.802 (15528337.961)	Up	1.54	0.8980 (W)
276_720.590	21641100.538 (42829750.192)	16286051.158 (29294918.065)	Down	-1.33	0.9171 (W)
277_787.668	66471093.321 (266936323.160)	1544058.308 (2608858.644)	Down	-43.05	0.9241 (W)
290_814.687	2995476.012 (3666486.007)	7308340.845 (28955751.380)	Up	2.44	0.9272 (W)
236_564.437	3430969.651 (6583981.089)	2944138.128 (5997249.350)	Down	-1.17	0.9332 (W)
27_482.324	186857662.261 (117291034.447)	176390046.194 (69218682.800)	Down	-1.06	0.9368 (W)
29_510.355	83472229.068 (70454977.232)	87358886.486 (80050080.325)	Up	1.05	0.9516 (W)
214_577.482	29793410.860 (48593424.493)	37941635.118 (50149605.726)	Up	1.27	0.9583 (W)
396_1278.526	1498956.239 (1898490.203)	1334026.724 (1199051.611)	Down	-1.12	0.9591 (W)
435_110.071	1696383.311 (4109225.348)	1142560.488 (1326378.186)	Down	-1.48	0.9599 (W)
422_283.107	5831453.780 (7672094.677)	7138714.416 (9610733.023)	Up	1.22	0.9665 (W)
238_135.079	3061000.941 (3066380.953)	2910620.989 (2354709.350)	Down	-1.05	0.9757 (W)
273_1230.145	1113629.943 (1078342.169)	1061390.046 (824922.577)	Down	-1.05	0.9757 (W)
423_1211.122	1374422.908 (1026476.081)	1464917.836 (1495351.703)	Up	1.07	0.9757 (W)
474_1414.530	1269284.821 (1080390.264)	1226286.409 (868285.873)	Down	-1.04	0.9757 (W)
523_549.416	1091231.500 (806657.252)	1741578.642 (5071678.600)	Up	1.6	0.9757 (W)
530_1091.706	1083883.252 (759420.610)	1179519.440 (1232452.954)	Up	1.09	0.9757 (W)
128_815.697	3235389.036 (6798176.130)	12835911.387 (64968464.267)	Up	3.97	0.9785 (W)
13_774.563	44598201.375 (62346053.530)	356918839.529 (525219542.261)	Up	8	0.9910 (W)
288_1385.099	1421833.732 (2352280.493)	1212727.142 (1239247.678)	Down	-1.17	0.9919 (W)
363_1254.308	1152132.932 (1071087.878)	1283255.767 (1914321.119)	Up	1.11	0.9919 (W)
376_1329.814	1518054.425 (1080615.152)	1543228.766 (1215176.866)	Up	1.02	0.9919 (W)
392_1233.503	1447034.646 (1211272.154)	1425738.586 (1096740.519)	Down	-1.01	0.9919 (W)
466_1488.004	1250893.490 (1376853.796)	1173930.611 (946957.715)	Down	-1.07	0.9919 (W)
473_1166.602	1186615.158 (1433505.188)	1111806.232 (1092304.487)	Down	-1.07	0.9919 (W)
365_1359.044	1262825.436 (1369505.405)	1189428.199 (914871.632)	Down	-1.06	1.0000 (W)
529_774.601	8541039.436 (47205625.556)	3374089.892 (8731592.161)	Down	-2.53	1.0000 (W)

Supplementary Table S3. Performance of regression models in Discovery group following 10-fold Cross Validation

**Metabolite algorithms**	**AUC (95% CI)**	**Sensitivity**	**Specificity**
D-erythro-C18-Sphingosine + Oleoyl L-carnitine +	0.984 (0.940- 1.000)	100%	96.8%
Palmitoyl ethanolamide + D-erythrosphingosine +	0.975 (0.944-1.000)	98.0 %	96.0 %
N-Palmitoylethanolamine + D-erythrosphingosine + Sphingosine 1-phosphate + Phenylalanyl tryptophan	0.965 (0.925-1.000)	94.0 %	98.0 %

Supplementary Table S4. Performance of metabolite + clinical factors regression models in Validation group following 10-fold Cross Validation

Metabolite + clinical predictor algorithms	AUC (95% CI)	Sensitivity	Specificity
D-erythro-C18-Sphingosine + Oleoyl L-carnitine + Previous Ectopic Pregnancy + Previous Adnexal Surgery	0.966 (0.930- 0.980)	96.8%	94.5%
Palmitoyl ethanolamide + D-erythrosphingosine + Previous Ectopic Pregnancy + Previous Adnexal Surgery	0.935 (0.912- 0.955)	95.0 %	93.0 %
N-Palmitoylethanolamine + D-erythrosphingosine + Sphingosine 1-phosphate + Phenylalanyl tryptophan + Previous Ectopic Pregnancy + Previous Adnexal Surgery	0.905 (0.885-0.920)	88.0 %	92.0 %
